# Ecosystem Services Related to Carbon Cycling – Modeling Present and Future Impacts in Boreal Forests

**DOI:** 10.3389/fpls.2019.00343

**Published:** 2019-03-26

**Authors:** Maria Holmberg, Tuula Aalto, Anu Akujärvi, Ali Nadir Arslan, Irina Bergström, Kristin Böttcher, Ismo Lahtinen, Annikki Mäkelä, Tiina Markkanen, Francesco Minunno, Mikko Peltoniemi, Katri Rankinen, Petteri Vihervaara, Martin Forsius

**Affiliations:** ^1^Finnish Environment Institute (SYKE), Helsinki, Finland; ^2^Finnish Meteorological Institute (FMI), Helsinki, Finland; ^3^Department of Forest Sciences, University of Helsinki, Helsinki, Finland; ^4^National Resources Institute (LUKE), Helsinki, Finland

**Keywords:** forest growth, carbon sink, vegetation active period, JSBACH, PREBAS, model, continuous monitoring, webcam

## Abstract

Forests regulate climate, as carbon, water and nutrient fluxes are modified by physiological processes of vegetation and soil. Forests also provide renewable raw material, food, and recreational possibilities. Rapid climate warming projected for the boreal zone may change the provision of these ecosystem services. We demonstrate model based estimates of present and future ecosystem services related to carbon cycling of boreal forests. The services were derived from biophysical variables calculated by two dynamic models. Future changes in the biophysical variables were driven by climate change scenarios obtained as results of a sample of global climate models downscaled for Finland, assuming three future pathways of radiative forcing. We introduce continuous monitoring on phenology to be used in model parametrization through a webcam network with automated image processing features. In our analysis, climate change impacts on key boreal forest ecosystem services are both beneficial and detrimental. Our results indicate an increase in annual forest growth of about 60% and an increase in annual carbon sink of roughly 40% from the reference period (1981–2010) to the end of the century. The vegetation active period was projected to start about 3 weeks earlier and end ten days later by the end of the century compared to currently. We found a risk for increasing drought, and a decrease in the number of soil frost days. Our results show a considerable uncertainty in future provision of boreal forest ecosystem services.

## Introduction

Ecosystem services (ES) are defined as the contributions that ecosystems make to human wellbeing (e.g., Costanza et al., 1997; Daily and Matson, 2008). The ES concept has become widely used and serves to emphasize the dependency of human society’s welfare on natural ecosystems (MEA, 2005; Costanza et al., 2017). A wide variety of ES are found in forests, which cover about 30% of global terrestrial area (FAO, 2018). Forests provide timber, and food, they conserve biodiversity, regulate water resources, and provide recreational opportunities (Shvidenko et al., 2005; Saastamoinen et al., 2014). Forests are essential factors of the global carbon (C) cycle, with an important role in regulating atmospheric concentrations of carbon dioxide (CO_2_) (Kurz et al., 2013). Boreal forests represent 29% of global forests (Shvidenko et al., 2005), comprising the circumpolar vegetation zone of high northern latitudes that covers one of the world’s largest biogeoclimatic areas (Brandt et al., 2013). The ES provided by boreal forests thus benefit human society both locally and on the global scale (Gauthier et al., 2015). Boreal forests account for about 20% of the global C sinks (Pan et al., 2011). Thereby boreal forests provide a climate regulating ES with bearing on climate change mitigation on the global level, although trade-offs are also recognized (Luyssaert et al., 2018).

Estimates of recent trends in above-ground biomass C indicate an increase for boreal forests (Liu et al., 2015). The stand and landscape level characteristics of the boreal forests of North America, Fennoscandia and Russia vary between the regions because of historical and current regional differences in natural disturbances and management practices (Gauthier et al., 2015; Kuuluvainen and Gauthier, 2018). Statistics from two neighboring boreal countries show increasing trends in roundwood increment in Finland (Vaahtera et al., 2018) and Sweden (Skogsdata, 2018). The future rate of C uptake by forests depends on how ambient temperature, land use and resource management practices evolve (Ahlström et al., 2012; Shao et al., 2013; Lappalainen et al., 2016). Rapid climate warming projected for the boreal zone (IPCC, 2013) has been observed in North America (McKenney et al., 2006; Price et al., 2013), Fennoscandia (Mikkonen et al., 2015; Hedwall and Brunet, 2016), and Russia (Schaphoff et al., 2016), which may have significant consequences for future boreal forest ES. Management practices may interact with climate change with consequences for forest biodiversity (Tremblay et al., 2018; Cadieux et al., 2019). Schaphoff et al. (2016) found that the impacts of climate change on Russia’s boreal forests are often superimposed by other environmental and societal changes, while Kalliokoski et al. (2018) estimated likely increases in future forest productivity in Finland, although with a high uncertainty. Climate change may affect the occurrence and extent of natural disturbances, such as insect outbreaks and fire (Schaphoff et al., 2016), e.g., Navarro et al. (2018) report a northward shift of spruce budworm (*Choristoneura fumiferana*) during the 20th century. Earlier thermal growing season has been found to be associated with earlier onset of biospheric C uptake, whereas earlier termination of biospheric activity was associated to later termination of thermal growing season (Barichivich et al., 2012). Warmer springs have consequences for bird reproduction (Ludwig et al., 2006; Wegge and Rolstad, 2017) and for moth multivoltinism, in combination with warmer summers (Pöyry et al., 2011). Less severe winter colds may promote the expansion of forest pests (Fält-Nardmann et al., 2018). Warmer winters may also have impacts on the possibilities for winter harvest on drained peatlands (Hökkä et al., 2016) and for winter recreation (Neuvonen et al., 2015).

The pathway from ecosystems, their biophysical structure, processes and functions to their benefit and value to human society, is illustrated by the ES cascade model, which portrays ES as emerging from the functional and structural properties of the ecosystem (Potschin and Haines-Young, 2011). In the Common International Classification of ES (CICES), services are classified into provisioning, regulating and maintenance, and cultural services (Haines-Young and Potschin, 2018). Several studies have discussed mapping of ES provision potential at different scales (Burkhard et al., 2009; Vihervaara et al., 2010; Maes et al., 2013; Albert et al., 2016a). The European Commission seeks to improve the basis for implementing the EU Biodiversity Strategy for 2020 (European Commission, 2011) by encouraging national ES assessments of the member states of EU through its flagship project MAES (European Commission, 2018). National accounting of natural capital needs information from national ES accounts and ongoing development of natural-capital accounts also supports national ES assessments (Schröter et al., 2016). Saastamoinen et al. (2014) applied the CICES hierarchy on the boreal forest ES in Finland, reporting their results in a conceptual and historical context. Mononen et al. (2016) developed a framework of ES indicators for Finland that complies with both national circumstances and international typologies such as the cascade model (Potschin and Haines-Young, 2011) and the CICES framework (Haines-Young and Potschin, 2018). ES are increasingly used to inform policies, and frameworks including ES analysis for decision support are becoming available (Albert et al., 2016b). Potentially highly promising avenues for underpinning resource allocation decisions are based on detailed place-based analyses of supply and demand of ES (e.g., Kopperoinen et al., 2014; Vauhkonen and Ruotsalainen, 2017). On the other hand, broad, unspecified ES may be more easily adopted by policy actors, according to Van Oudenhoven et al. (2018).

Our aim is to illustrate potential impacts of climate change on boreal forest ES. In this paper we (i) apply two dynamic ecosystem models (JSBACH and PREBAS) driven by a set of climate change scenarios to simulate present day and future values of a set of biophysical variables; (ii) compare simulated present day values with available observations and statistical data; (iii) suggest interpretations of the biophysical variables as ES; (iv) discuss the consequences of changes in these biophysical variables on future ES of boreal forests (Supplementary Figure 1).

## Materials and Methods

### Ecosystem Models

We applied the land ecosystem model JSBACH (Raddatz et al., 2007; Reick et al., 2013; Goll et al., 2015; Gao et al., 2016) and the stand growth model PREBAS (Valentine and Mäkelä, 2005; Peltoniemi et al., 2015b; Minunno et al., 2016, 2019) for the land area of mainland Finland, using input data in spatial resolution ranging from 16 m (forest resources, PREBAS), 0.1° (land surface characteristics, JSBACH) to a 0.2° × 0.1° longitude-latitude grid (climate data). We report simulated results for a set of biophysical variables for four time periods (1981–2010, 2011–2040, 2041–2070, 2071–2100). Two variables reflect directly ecosystem C fluxes: gross primary productivity (GPP; gCm^-2^ yr^-1^) and net ecosystem exchange (NEE; gCm^-2^ yr^-1^). Ecosystem phenology is described by three variables: the length of vegetation active period (VAPlength; days), when terrestrial vegetation is assimilating C through photosynthesis, the start (end) of vegetation active period (VAPstart; VAPend; days from January 1st, when the daily level of photosynthesis first exceeds, or returns to below 15% of its summertime value). Stemwood growth (m^3^ yr^-1^) was simulated with PREBAS, and the number of summer dry days (soil moisture < 5th percentile of reference period) and number of winter days with soil frost were simulated with JSBACH. The models have been compared earlier and found to produce similar GPP estimates at local and national level (Peltoniemi et al., 2015a). Here we use the two models in parallel to provide further information about the changes in biophysical variables under climate change, and also in supplementing each other for variables predicted by only one of the models. We present the results as boxplots and tabulated percentile values, and cumulative distribution functions aggregated over the whole area, as well as time series and maps for some variables.

JSBACH is a land surface model of an earth system model of Max Planck institute for meteorology (MPI-MET) and describes the biogeophysical processes that regulate the balances of water and CO_2_. The storage of water and C into the ecosystem as well as their release to the atmosphere is regulated by the climatic variables. Land vegetation is divided into plant functional types (PFT), and for our domain (the Finnish mainland) we based the PFT distribution on Finnish CORINE land cover data (CLC, 2012), which contains information about soil type, thus providing soil characteristics consistent with the vegetation cover data. Seasonal development of leaf area index is regulated by air temperature and soil moisture with PFT specific maximum leaf area index as a limiting value. For the generation of the seasonal cycle, PFTs are divided in summergreen, evergreen, grass and crop phenology types (Böttcher et al., 2016). Photosynthesis is described according to Farquhar et al. (1980), using PFT-specific parameters for the maximum carboxylation (*V*_max_) and electron transport (*J*_max_) rates. Global parameter values were used in this study, and as JSBACH was run without explicit nitrogen cycle, the mean values at 25°C (*V*_max_*25*) were applied, with *J*_max_*25* = 1.9 ⋅*V*_max_*25* for all PFTs (Wullschleger, 1993; Kattge et al., 2009). The photosynthetic rate is resolved first under non-water-stressed conditions to attain photosynthetic activity (Schulze et al., 1994), and limitations in water availability are accounted for Knorr and Heimann (1995) and Knorr (2000). The radiation absorption within the vegetation canopy is calculated for three layers (Sellers, 1985), accounting for clumping of the leaves in sparse canopies (Knorr, 2000). In addition to the canopy processes the model consists of a 5-layer soil moisture description (Hagemann and Stacke, 2015) and the Yasso soil C module (Goll et al., 2015). We adopted a formulation that delays the beginning of photosynthetic activity of evergreen species in spring (Kolari et al., 2007), as according to Böttcher et al. (2016) the start date of the photosynthetically active season of coniferous evergreens in the model is ahead of the observed. We decreased the threshold of the temperature sum regulating the bud-break from 4 to 2°C in accordance to findings by Böttcher et al. (2016). Furthermore, a condition that reduces stomatal conductance under supersaturation was removed because it falsely prohibits photosynthesis under conditions of very high humidity.

In addition, simulations were performed with PREBAS, a C-balance based stand growth and gas exchange model (Valentine and Mäkelä, 2005; Peltoniemi et al., 2015b; Minunno et al., 2019). In PREBAS, photosynthesis (GPP) and evapotranspiration are calculated using a light-use-efficiency approach linked to soil moisture and driven by daily environmental inputs of radiation, temperature, vapor pressure deficit, precipitation and ambient CO_2_ concentration (Peltoniemi et al., 2015b; Minunno et al., 2016; Kalliokoski et al., 2018). GPP is allocated to mean-tree growth and respiration at an annual time step, and allocation of growth to different tree components is based on conservation of structural constraints, e.g., the pipe model (Valentine and Mäkelä, 2005). Tree mortality due to crowding is included in the model. The growth module updates canopy leaf area index which is used as input to the gas exchange module in the following year. To calculate NEE, PREBAS has been linked with the soil C model YASSO07 through annual litter inputs (Liski and Westman, 1995; Tuomi et al., 2009). In addition to weather data, PREBAS requires information on the initial state of the simulated forest, and forest management actions, including thinning, clearcut and regeneration, to obtain a realistic dynamic pattern of forest development. The relatively simple structure of the model allows us to apply it at a large regional scale and make simulations of C and water fluxes that are both climate and management sensitive. Here, we defined forest management on the basis of current management recommendations in Finland (Sved and Koistinen, 2015; Minunno et al., 2019). The simulations were carried out by linking PREBAS to information on soil fertility, stand basal area, mean height and mean diameter, derived from 16 m resolution multisource forest inventory data maps (Tomppo et al., 2014; Mäkisara et al., 2016; LUKE, 2018a,b). The forest resource maps were divided into 16 km grid cells. Within each grid cell, simulations were carried out for 50 forest classes with the highest proportional areas, accounting also for the remaining classes by distributing their area to these dominant classes. The forest classes represented different combinations of dominant tree species, age, basal area and mean height. Categories of soil fertility were herb-rich heath, mesic heath, sub-xeric heath, xeric heath, and poorer types (Cajander, 1949). To account for mineral and peat soil types, we used two sets of soil parameters, one describing typical mineral soil, and one for a soil layer with high soil water retention capacity (depth = 430 and 1000 mm, respectively), as PREBAS does not simulate organic soils as such. The simulated annual values for each class were aggregated to grid cell level based on 16 m resolution forest maps. Climate data from the nearest climate grid point were used for each 16 km forest simulation grid cell.

We compared the simulated annual NEE for the period 1981–2010 with reported values of the C sink of Finnish forests (Official Statistics of Finland, 2019). The simulated stemwood growth values for the period 2009–2012 were compared to observed forest growth for the same period. The simulated VAPstart days were compared to estimates based on webcam images (Arslan et al., 2017; Peltoniemi et al., 2018a,b), as well as to those estimated from satellite observations (SYKE Vegetation Phenology 2001–2017).

### Climate Change Scenarios

**FIGURE 1:**
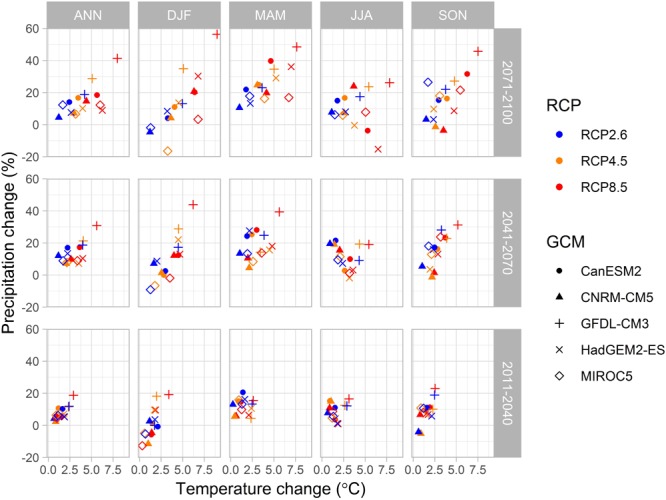
Annual and seasonal projected change in precipitation sum (%) versus change in mean temperature (°C) in Finland. Changes calculated from the reference period 1981–2010 to periods 2011–2040 (bottom), 2041–2070 (middle) and 2071–2100 (top) from regionally down-scaled data from simulations with three levels of climate forcing (RCP) driving five down-scaled CMIP5 global climate models (GCM). Seasons ANN annual (left), DFJ winter, MAM spring, JJA summer, and SON autumn (right).

The climate change scenario data through years from 1981 to 2100 were obtained as the output from five global climate models (GCMs; CanESM2, CNRM-CM5, GFDL-CM3, HadGEM2-ES and MIROC5) from the fifth phase of the Coupled Model Intercomparison Project (CMIP5; Meehl et al., 2009; Taylor et al., 2012). We used output for climate models driven by three representative concentration pathways (RCPs), that lead to radiative forcing levels of 2.6, 4.5 and 8.5 W m^-2^ by the end of the century (Moss et al., 2010; Van Vuuren et al., 2011). These pathways include one mitigation scenario (RCP2.6), one stabilization scenario (RCP4.5) and one high emission scenario (RCP8.5) (Van Vuuren et al., 2011). The resulting climate variables were down-scaled to a 0.2° × 0.1° longitude-latitude grid by a quantile-quantile type bias correction algorithm for daily mean temperature, relative humidity, shortwave radiation and wind speed (Räisänen and Räty, 2013) and parametric quantile mapping for daily precipitation (Räty et al., 2014). Gridded harmonized FMI meteorological data by Aalto et al. (2013) were used. Long-wave radiation data were interpolated by a bilinear interpolation method. Global mean CO_2_ concentrations from the RCPs 2.6, 4.5 and 8.5 were linearly interpolated to monotonously increase through the calendar years. The changes of temperature and precipitation from a baseline period 1981–2010 to periods 2011–2040, 2041–2070, and 2071–2099 in Finland varied considerably between data from five down-scaled CMIP5 climate models (Figure 1). PREBAS was run with all three RCPs, while JSBACH input was taken from RCP4.5 and RCP8.5. The RCP8.5 output of HadGEM2-ES was not included in JSBACH runs.

### Ecosystem Services

On the basis of the analysis of Saastamoinen et al. (2014) and Mononen et al. (2016), we identified a set of key boreal forest ES. In the provisioning services section of the CICES classification, we selected productivity and supply of harvestable wood as key benefits to human society (Table 1). Forestry land covers 86% of the total land area of Finland, and forest industry products accounted for 20% of the total value of Finnish goods exports in 2017 (Vaahtera et al., 2018). Gross primary production, or the rate at which terrestrial vegetation assimilates CO_2_ in photosynthesis, (GPP gC m^-2^ yr^-1^) was simulated by JSBACH and PREBAS, and forest stemwood growth (m^3^ yr^-1^) by PREBAS.

**Table 1:** Ecosystem services with corresponding biophysical proxies.

Section	Class	Function	Benefit (B), or Loss (L)	Modeled results of biophysical variables
Provisioning services	Wood	Growing stock increment	Productivity (B), Supply of harvestable wood (B)	GPP (gCm^-2^ yr^-1^), Stemwood growth (m^3^ ha^-1^ yr^-1^)
Regulation and maintenance services	Climate regulation	Carbon sequestration	CO_2_ forcing (L)	NEE (gCm^-2^ yr^-1^)
	Transpiration	Evapotranspiration	Summer drought (L)	Number of dry summer days
	Soil structure	Bearing capacity	Opportunities for winter harvest (B)	Number of soil frost days
	Maintaining habitats	Reproduction	Viable populations (B/L)	VAPstart, VAPend, VAPlength (days)
Cultural services	Nature tourism	Natural events and phenology	Opportunities for recreational experiences (B/L)	VAPstart, VAPend, VAPlength (days)

For the regulation and maintenance section of the CICES classification, the key service studied was related to regulating climate through C sequestered by growing vegetation. The corresponding benefit to society was avoided CO_2_ forcing (Table 1). The proxy for CO_2_ forcing, NEE (gC m^-2^ yr^-1^), is the difference between the total respiration rate of the ecosystem, and GPP. When the flux is from the atmosphere to the ecosystem, NEE is negative, and when the flux is from the ecosystem to the atmosphere, NEE is positive. In 2017, the Finnish total greenhouse gas emissions were 55.5 million metric tons CO_2_ eq, including energy, industrial, agriculture, and waste sectors. In contrast, the net removal from the atmosphere by forest land was 27.0 million metric tons CO2 eq. Altogether, the net sink of the land use, land use change, and forestry sector (LULUCF) was 20.4 million metric tons CO_2_ eq (Official Statistics of Finland, 2019). Climate change impacts on future values of NEE were simulated by JSBACH and PREBAS.

Risk of productivity losses related to summer drought, and decreasing opportunities for winter harvest on drained peatlands were considered as examples of regulating services. JSBACH simulations of the number of dry summer days, and soil frost days, were used for the climate impact projections. We used the length and the timing of the vegetation active period as proxies for both a regulating service (the maintenance of habitats), and a cultural service (recreational opportunities). The impact of climate change on the length (VAPlength, number of days) and timing of the vegetation active period (VAPstart, VAPend, day of year) was simulated with JSBACH and PREBAS.

Current potential ES provision was estimated as the median values of the simulated biophysical variables for the reference period (1981–2010). The impact of climate change on ES provision was interpreted as future changes in simulated biophysical variables compared to reference period median values. Positive changes in GPP, stemwood growth and number of soil frost days are in our analysis associated with benefits for human society. Positive values of NEE represent a flux from the ecosystem to the atmosphere (CO_2_ emissions), while negative values of NEE (CO_2_ removals) represent avoided CO_2_ forcing. Thus, in our analysis, positive changes in the simulated values of NEE are considered losses, and negative changes benefits to human society. Increasing frequencies of dry summer days are similarly considered losses. An increase in the length of the vegetation active period, however, may lead to both benefits and losses.

## Results

### Provisioning Services: GPP and Supply of Harvestable Wood

**FIGURE 2:**
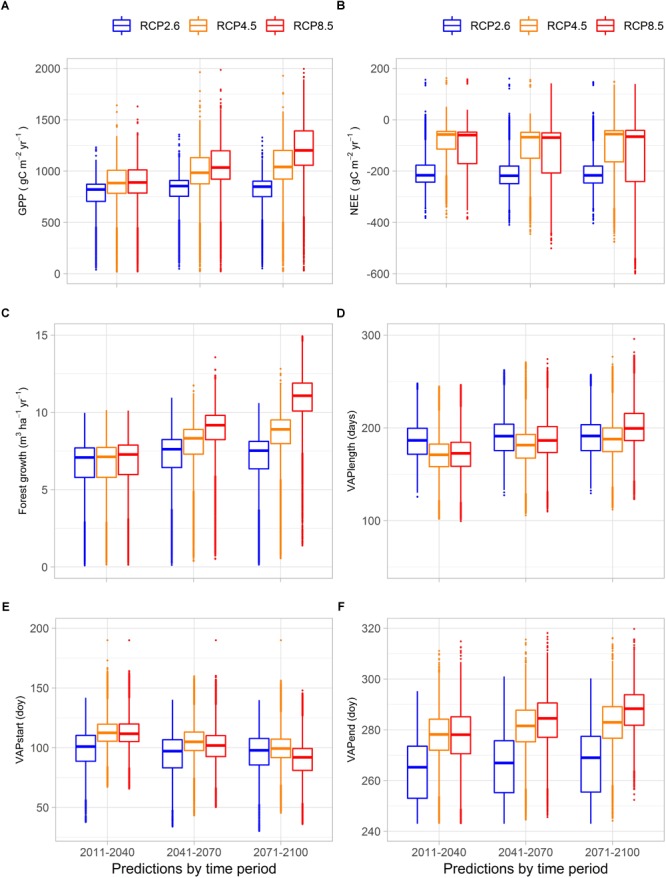
Summary of simulated values for **(A)** GPP (gC m^-2^ yr^-1^); **(B)** NEE (gC m^-2^ yr^-1^); **(C)** forest growth (m^3^ ha^-1^ yr^-1^); **(D)** VAPlength (days); **(E)** VAPstart (days from January 1st); **(F)** VAPend (days from January 1st) for three time periods (2011–2040; 2041–2070; 2071–2100). The results are obtained from simulations with PREBAS and JSBACH using future climate data from five climate models with driving scenarios RCP2.6, RPC4.5 and RCP8.5 (PREBAS), and RCP4.5 and RCP8.5 (JSBACH). Forest growth based on PREBAS results only.

**FIGURE 3:**
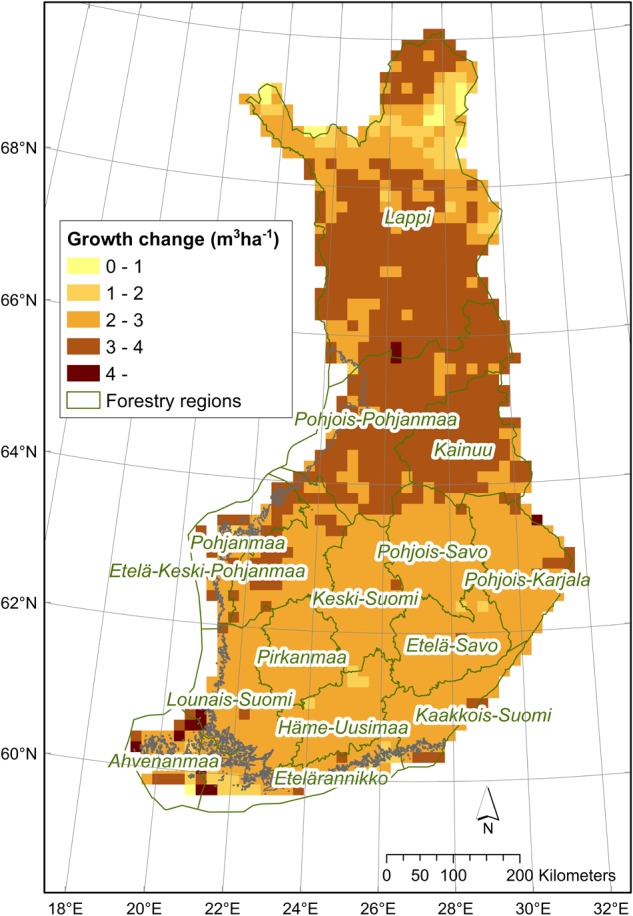
Mean modeled change in annual growth (m^3^ ha^-1^ yr^-1^) from 1981–2010 to 2041–2070, calculated over all climate change scenarios. Forestry regions and their borders indicated on the map.

GPP was projected to increase with climate change, more so with higher radiative forcing (RCP8.5), and more toward the end of the century (Figure 2). For the low emission scenario (RCP2.6) hardly any difference between the time periods can be seen in the simulated GPP values. Differences between the medium and the high emission scenario simulations (RCP4.5 and RCP8.5) are evident only from the mid-century onward (Figure 2 and Supplementary Figure 2). The estimated median increase in GPP was 34% for the mid-century time period (2041–2070), and 46% for the end of the century (2071–2100), over all radiative forcing levels (Supplementary Table 1). Current observed mean annual growth in 15 forestry regions of Finland compared well (*R*^2^ = 0.95) with PREBAS simulated results for the same period (2.0–7.6 m^3^ ha^-1^ yr^-1^; Supplementary Figure 3). In the climate change projections for mean annual forest growth, all scenarios give similar results for the first time period (2011–2040, Figure 2 and Supplementary Figure 4), whereas the differences become clear later, especially toward the end of the century. The highest increase in mean annual growth was projected for the north of Finland (Figure 3). The simulated country-wide median forest growth increased from 5.6 m^3^ ha^-1^ yr^-1^ in 1981–2010 by 2.7 to 8.3 m^3^ ha^-1^ yr^-1^ in 2041–2070, and by 3.4 to 8.7 m^3^ ha^-1^ yr^-1^ in 2071–2100 (Supplementary Table 1), corresponding to an increase of 48% by mid-century, and 60% by the end of the century.

### Regulating Services

#### Avoided CO_2_ Forcing

Interpreted as an ES, a more negative value of NEE means that the ecosystems of the region have a potential to mitigate the radiative forcing by atmospheric CO_2_, which is the most important contributor of human induced climate change. Across Finland, the simulated median annual NEE was –50 gC m^-2^ yr^-1^ for the period 1981–2010 (Supplementary Table 1). Reported current national total annual C sink of forest biomass and soil varied in the range –35 to –18 Mt CO_2_ eq yr^-1^ in the period 1990 to 2017 (Official Statistics of Finland, 2019). Averaged over total forestry land (262,000 km^2^), the reported range corresponds to annual removals of –36 to –19 gC m^-2^ yr^-1^. Our simulated reference period annual C sink thus clearly exceeds the range of reported current values (Official Statistics of Finland, 2019).

Simulations with the low emission scenario (RCP2.6), which include only PREBAS results, represent clearly larger C sink values (more negative NEE) than simulations with the other scenarios, for all time periods. The high emission scenario (RCP8.5) shows the largest range in simulated NEE results (Figure 2). In projections across Finland from both ecosystem models and all climate scenarios, the median removal of CO_2_ increased with 48 and 39% by mid-century and the end of the century, respectively. The corresponding simulated median values were NEE –74.1 and –66 gC m^-2^ yr^-1^ for the period 2041–2070 and 2071–2100 (Supplementary Table 1).

#### Increasing Risk for Drought and Decreasing Opportunities for Winter Harvest

Warming temperatures, changing precipitation patterns (Figure 1) and increasing forest growth (Figure 2, 3) altered the water availability in the JSBACH simulations. The risk for drought was slightly increasing for all scenarios. Much of the change is due to earlier snow melt and thus possibility for earlier drought events. Decreasing precipitation was projected for some climate models in winter for all time periods, and also in summer for the end of the century (Figure 1). The mean number of dry summer days was approximated to around 4 days for the reference period. By the end of the century (2071–2100), the mean simulated number of dry summer days increased to about 15 days in the south and to 10 days in the north (Supplementary Table 2). In southern Finland, simulations for the mid-century gave even higher number of dry days (23). In the JSBACH simulations, the number of soil frost days was decreasing in all simulations (Supplementary Table 2). The changes were largest in southern Finland under the high forcing scenario (RCP8.5), from 134 days in 1981–2010 to 25 days in 2071–2100. This means the opportunities for winter harvest on drained peatlands are decreasing with all scenarios, more in the south than in the north.

### Regulating and Cultural Services: Maintaining Habitats, Nature Tourism

**FIGURE 4:**
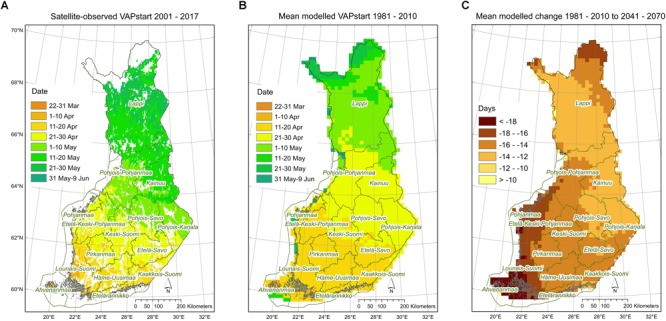
Start of vegetation active period **(A)** from satellite observations 2001–2017, calculated as weighted average for all forest types based on their proportions in the satellite grid – days from January 1st expressed as dates; **(B)** as mean modeled results from JSBACH and PREBAS 1981–2010 – days from January 1st expressed as dates; and **(C)** mean modeled number of days difference in 2040–2070 compared to 1981–2010.

Estimates of the start dates of the vegetation active period on the basis of webcam images were similar to those simulated by the JSBACH model (see section “Webcam Network” in Supplementary Material and Supplementary Figure 6). The vegetation active period ended slightly later according to the JSBACH results than based on the webcam images. VAPstart in Finland occurred in median on 28 April in all simulations for the present period (1980–2010) (Supplementary Table 1). In comparison, for the period 2001–2017, VAPstart in Finland was determined from satellite observations (SYKE, 2018). The satellite-derived median VAPstart occurred on 13 April in evergreen coniferous forest. JSBACH simulations included all PFTs whereas PREBAS simulations included forested areas only. Thus, the simulated present day VAPstart seems to be slightly ahead of satellite observations with a median VAPstart on 5 May for all forest types (Figure 4). In comparison to reference conditions, the vegetation active period started (VAPstart) about 2 weeks earlier in the mid-century, and 3 weeks earlier toward the end of the century. This means spring would start earlier in April than at present. The vegetation active period ended (VAPend) by the end of the century in early October, compared to late September in the reference conditions (Figure 2 and Supplementary Table 1). In the climate change simulations, the length of the vegetation active period (VAPlength) increased from 162 days with about 20 days in the mid-century and with 30 days by the end of the century (Figure 2 and Supplementary Table 1).

## Discussion

### Provisioning Services: GPP and Supply of Harvestable Wood

We aggregated the results from the two ecosystem models in order to account for the uncertainty inherent in the use of different assumptions, e.g., JSBACH simulations do not account for forest management. In this paper we focus on presenting the results on the whole-country level. Data on grid-based results of the biophysical variables for each ecosystem model and each climate scenario separately are available online (Metadata, 2018a,b). These online metadata descriptions include links to the Climateguide.fi service, where maps of the grid-based results can be found by selecting the category “Terrestrial ecosystems,” and the variable of interest “GPP,” “NEE,” etc. The maps are displaying results from each ecosystem model (JSBACH, PREBAS), climate model, and climate forcing level RCP separately. Seasonal patterns of GPP simulated by PREBAS and JSBACH aggregated to the national level were almost identical, and temporal trends of annual totals were also parallel. This is remarkable in light of earlier results, as ecosystem models tend to vary quite much in their GPP estimates globally and in the northern latitudes (Anav et al., 2013; Shao et al., 2013). However, it has to be noted that the current version of the JSBACH photosynthesis model has been modified for boreal conditions using partly the same empirical evidence as PREBAS (e.g., Mäkelä et al., 2004; Kolari et al., 2007).

The climate change simulations of forest growth did not account for growth restrictions due to limited access to nutrients, mainly nitrogen or phosphorus. Forest growth in higher CO_2_ concentrations may become nutrient limited in nutrient deficient sites (e.g., Wieder et al., 2015). As PREBAS projections of future growth were made assuming current forest practices continue in the future (Sved and Koistinen, 2015; Minunno et al., 2019), here no provision was made for potential adaptive responses by the forest owners, e.g., aiming for more variation in stand structure, stand age or silvicultural management (Blennow, 2012; Montoro Girona et al., 2017), introducing new tree species or varieties (Laakkonen et al., 2018; Sousa-Silva et al., 2018), or striving to maximize positive synergies between ecosystem services (Kuuluvainen and Gauthier, 2018). Current total volume of growing tree stock in Finland is 2.5 billion m^3^ with a total annual increment of 107 million m^3^, which corresponds to annual average 6.8 and 3.2 m^3^ ha^-1^ in southern and northern Finland, respectively. Total volume of roundwood harvested from forests was a record high 72.4 million m^3^ in 2017, about 17% higher than the average of the preceding ten-year period (Vaahtera et al., 2018). Current forest policies in Finland aim to increase the annual cut to over 80 million m^3^ in the next decades. Our simulations indicate that these plans could be feasible in a changing climate, however, increased cutting levels would also imply a lower growing stock than that in our simulations which assumed continuing the current harvest intensity. This would have further implications on C sequestration that need to be explored separately. We did not study the impact of climate change and cutting regimes on biodiversity indicators, such as the amount of dead wood, but others have found significant effects of bioenergy extraction on dead wood levels (e.g., Hof et al., 2018) and conflicting objectives of wood production and biodiversity conservation (Mönkkönen et al., 2014; Angelstam et al., 2019). In their comprehensive review on the retention approach for forestry, Lindenmayer et al. (2012) argued that the practice of permanently retaining significant elements of the original forest is crucial for maintaining multiple forest values, such as biodiversity and carbon stocks. Recently, partial harvest methods were found adequate for regeneration for sustainable forest management in Canadian boreal forests (Montoro Girona et al., 2017, 2018).

### Regulating Services

#### Avoided CO_2_ Forcing

Regarding NEE, JSBACH and PREBAS models produced fairly different patterns especially under climate change – PREBAS predicted more negative NEE values than JSBACH (MONIMET, 2017; Peltoniemi et al., unpublished results). We assume that the differences are caused by different approaches to describing tree growth and management in these models, JSBACH assuming steady-state conditions and PREBAS accounting for forest growth and management explicitly. The harvests lead to a drain of stemwood from the forest which reduces the amount of C entering the soil as coarse woody debris, and therefore reduces the rate of heterotroph respiration from the soil. In our presentation, the NEE projections aggregate results from these models with different assumptions concerning vegetation and forest management, and the variance of the results reflect uncertainties in process descriptions and parameterizations. Forests remain a C sink in our simulation results.

It has been shown that future development of NEE varies much according to ecosystem model and applied climate forcing. According to Ahlström et al. (2012), simulations with a dynamic global vegetation model (LPJ-GUESS), forced by 18 CMIP5 climate projections, showed net release by 2100 for the boreal region. However, the projections were not in full agreement with each other. The magnitude of the release varied, and even turned to uptake in some simulations. Further, according to an ensemble of CMIP5 models both uptake and release of C by land ecosystems is accelerated in the twenty-first century (Shao et al., 2013). The boreal latitudes could become a major C sink by 2100 in many models. It should also be recognized that there is a trade-off situation between the ES supply of “harvestable wood” and “avoided radiative forcing.” According to calculations by the Finnish Climate Change Panel, meeting the Finnish targets for implementing the Paris climate change agreement would, in addition to stringent GHG emission reductions, also require a large increase in the C sinks (Finnish Climate Change Panel, 2018). Similar measures would be required also at the global level (Rockström et al., 2017; Rogelj et al., 2018). Intensification of biomass removals from forests may invoke harmful impacts on forest productivity, biodiversity, soil quality, and climate change mitigation potential (Aherne et al., 2012; Forsius et al., 2016; Mäkelä et al., 2016; Soimakallio et al., 2016; Vanhala et al., 2016; Eyvindson et al., 2018).

#### Increasing Risk for Drought and Decreasing Opportunities for Winter Harvest

In boreal and northern conditions, dry and warm summers have been found to increase drought-related symptoms in trees, such as defoliation or discoloration (Muukkonen et al., 2015), limit tree radial growth (Aakala and Kuuluvainen, 2010) and contribute to severe disturbances (Aakala et al., 2011). Satellite-derived estimates of vegetation growth trends indicate a clear negative response to recent dry summer conditions (Piao et al., 2011). Shorter periods of frozen soil increase wind damages to forest, as the trees are less firmly anchored in non-frozen soil. Wind-thrown trees may also serve as starting points for bark beetle outbreaks (Pukkala, 2018). In boreal forests, timber is mainly harvested with heavy machinery, which can operate only if the soil has a sufficient bearing capacity (Suvinen, 2006). Higher and more variable air temperatures in winter, coupled with longer and more frequent rain events, may cause pronounced edge effects by skidding trails (Montoro Girona et al., 2016), or lead to periods during which the sites on drained peatlands are not accessible (Kokkila, 2013).

### Regulating and Cultural Services: Maintaining Habitats, Nature Tourism

For regulating ES, earlier start of vegetation active period means earlier opportunities for birds, insects and other species, but is also linked to the risk for coincident occurrences of cold spells that may be detrimental. For cultural ES, climate warming is expected to lead to earlier opportunities for nature tourism linked to the coming of spring, such as bird watching and observing shoot growth, bud bursting and flowering. However, potential detrimental long-term impacts of climate change on species diversity and conservation are also well recognized (e.g., Thomas et al., 2013; Virkkala et al., 2013; Heikkinen et al., 2015; Virkkala, 2016; Cadieux et al., 2019). For regulating ES, later end of vegetation activity may mean decreased leaching of N in autumn. For provisioning and cultural ES, later end of vegetation may be associated with improved opportunities for the growth and picking of mushrooms and berries, although the success of these species is mostly regulated by local weather conditions.

In terms of regulating ES, this means that there is a positive impact on the reproduction and survival of birds, insects and other species that are dependent on forest vegetation activity. There might be negative impacts due to mismatch of animal production and food supply, or better match between insects and host plants and resulting damages that could alter ecosystem composition (Foster et al., 2013; Pureswaran et al., 2015). The spruce bark beetle (*Ips typographus*), a serious pest in boreal forests may benefit from warmer temperatures (Annila, 1969) and earlier occurrence of spring (Lange et al., 2006; Zang et al., 2015), increasing the risks for declining forest health (Blomqvist et al., 2018). With the lengthening of the vegetation active period, there is also the option for spatial migration of plant and animals to more northern areas (e.g., Jeganathan et al., 2014).

Summertime nature tourism in the Nordic countries, e.g., for fishing, biking, hiking, kayaking, bird and animal watching, was perceived as interesting activities by respondents in an international survey (MEK, 2010). According to the Finnish national outdoor recreation inventory, almost 70% of annual nature visits were made during the 5 months from May to September, the most popular outdoor recreation activities being walking, swimming, spending time in nature and at a recreational home, picking wild berries, cycling, boating, fishing, cross-country skiing and mushroom picking (Neuvonen and Sievänen, 2011). According to Lankia et al. (2015), the overall value of recreational nature visits was considerable in comparison to other land uses. It is likely that an increase in VAPlength would be beneficial to human society. It should, however, be noted that as the increase in VAPlength may be accompanied by increased probability of spring and autumn rains (Ruosteenoja et al., 2016), the recreational value may not be realized. Here, we do not report simulated future changes in number of snow cover days in response to climate change. It is clear, however, that the occurrence of earlier start of spring and later beginning of autumn season are coupled to shorter winter periods, leading to losses as regards the opportunities for winter tourism. Cross-country skiing is an important recreational activity in Finland (Neuvonen and Sievänen, 2011) and Neuvonen et al. (2015) presented an interactive tool to study the implications of climate change for cross-country skiing. A considerable proportion of recreational nature visits are made to nature conservation areas, and in the case these visits constitute an increased pressure on the nature conservation areas, the goals of conservation and recreation may be conflicting. It is more likely, however, that the goals of forestry and conservation are colliding. The recreational experience is not independent of the maintenance of habitats, and we are aware of the risk of double attribution in estimating quantitative values for these services, e.g., in monetary terms. It was, however, beyond the scope of our work to assess quantitative values for the services we have studied. In the case of the qualitative analysis carried out here, we think we are justified to use the vegetation active period characteristics to illustrate both the potential for habitat maintenance and recreation opportunities.

For the near decades, even in mid-century, the three emission levels (RCP) gave broadly similar projected temperature increase and change in precipitation on the annual and country-wide level (Figure 1). Different climate models, however, gave varying responses especially for seasonal changes. Extreme warming (>5°C) was projected by some climate models by the end of the century for the high emission scenario. Ruosteenoja et al. (2016) call the late century response to RCP8.5 “*an alarm signal, demonstrating the furious climatic changes that would be expected if the mitigation of greenhouse gas emissions were totally neglected.*” Although spatially explicit data have been used to drive the ecosystem models, the results we report here are mainly estimates aggregated over the whole country. We realize that important detailed information is lost in such an aggregation, especially concerning tradeoffs between ES, and the spatial distribution and balance of ES supply and demand (Potschin and Haines-Young, 2013; Vauhkonen and Ruotsalainen, 2017). The value of boreal forest future ES has also not been addressed in this paper. We have qualitatively described future ES on the basis of simulated biophysical quantities such as forest growth and CO_2_ exchange with the atmosphere. Analyzing current and future valuation of ES by forest owners and other stakeholders would be needed in order to introduce boreal forest ES into a natural capital framework (Schröter et al., 2016; Lai et al., 2018), or to assess forest carbon policies (Pohjola et al., 2018). Such considerations are, however, beyond the scope of our work.

## Conclusion

In our analysis, climate change impacts on key boreal forest ES are both beneficial and detrimental. Increased GPP is beneficial, leading to increased C sequestration, as well as increased forest growth and improved supply of harvestable wood. Warmer temperatures lead, however, also to higher ecosystem respiration, which in some instances cause increasing fluxes of CO_2_ from vegetation to atmosphere (NEE > 0). Although GPP is steadily increasing in our simulations, there are times in early and late 21st century when GPP is smaller than ecosystem respiration and annual NEE becomes positive. Here, the assumptions concerning future conditions included only different climate change scenarios, not considering any adaptation measures, e.g., regarding changing forestry practices. Thus, future forest growth simulations with PREBAS reflect a continuing business as usual response of the forestry sector. This means the current forestry practices are assumed to continue, and possible shifts toward higher harvest rates to accommodate increased demand for bioenergy have not been considered here. Possible increased risk of forest damage was also not accounted for, although increasing probabilities of more frequent insect outbreaks and wind throw damages have been recognized. There is still a large uncertainty in predicting future forest growth, since the models disregard natural disturbances, and are not constrained by potential nutrient limitations (N, P and base cations). These limitations call for further model developments as well as biomass and flux data to inform process based modeling. Our current simulation results likely represent the high end of the future growth estimates. We also noted the need to consider a balanced approach between forest harvesting, C sequestration potential and nature based ecosystem services in forested ecosystems.

While some of the studied ES are expected to improve with climate change (biomass growth, nature tourism), the projected decrease in number of soil frost days is expected to decrease the opportunities for winter harvest in forestry especially in the south. Future levels of net ecosystem exchange of CO_2_ are uncertain; some simulations indicate decreasing radiative forcing levels, while other simulations lead to increasing levels. Forests remain C sinks, however, in all simulations. The risk for summer drought is slightly increasing in the whole country. Climate warming is expected to lead to earlier opportunities for spring-time nature tourism, and may improve opportunities for autumn mushroom and berry picking. The opportunities for winter sports are decreasing while hiking opportunities are increasing with earlier occurrences of spring.

Our results indicate the range of uncertainty in future provision of boreal forest ES. The estimates described in this paper may provide input to comprehensive systems for integrated natural capital accounting, as well as sharing such information via hubs such as National Clearing House Mechanism for the Convention on Biological Diversity (Finnish Ecosystem Service Indicators, 2015). Furthermore, the examples in this paper may highlight the potential for advancing ES monitoring, such as improving information on ecosystem variables for instance by high-resolution Earth Observation or novel *in situ* observation networks such as webcams.

## Data Availability

The datasets generated for this study are available on request to the corresponding author.

## Author Contributions

MH planned and drafted the manuscript. MH, TA, AnA, AlA, TM, AM, FM, MP, KB, and KR planned the framework. TA and TM provided and interpreted JSBACH model results. AM, MP, and FM provided and interpreted PREBAS model results. IL provided the web interface of the model results. KB contributed revised material on phenology and performed GIS analysis. AnA, IB, KR, PV, and MF contributed and revised material on ecosystem services.

## Conflict of Interest Statement

The authors declare that the research was conducted in the absence of any commercial or financial relationships that could be construed as a potential conflict of interest.

## References

[B1] Aakala T. Kuuluvainen T. (2010). Summer droughts depress radial growth of *Picea abies* in pristine taiga of the Arkhangelsk province, northwestern Russia. *Dendrochronologia* 29 67–75. 10.1016/j.dendro.2010.07.001

[B2] Aakala T. Kuuluvainen T. Wallenius T. Kauhanen H. (2011). Tree mortality episodes in the intact *Picea abies*-dominated taiga in the Arkhangelsk region of northern European Russia. *J. Veg. Sci.* 22 322–333. 10.1111/j.1654-1103.2010.01253.x

[B3] Aalto J. Pirinen P. Heikkinen J. Venäläinen A. (2013). Spatial interpolation of monthly climate data for Finland: comparing the performance of kriging and generalized additive models. *Theor. Appl. Climatol.* 112 99–111. 10.1007/s00704-012-0716-9

[B4] Aherne J. Posch M. Forsius M. Lehtonen A. Härkönen K. (2012). Impacts of forest biomass removal on soil nutrient status under climate change: a catchment-based modelling study for Finland. *Biogeochemistry* 107 471–488. 10.1007/s10533-010-9569-4

[B5] Ahlström A. Schurgers G. Arneth A. Smith B. (2012). Robustness and uncertainty in terrestrial ecosystem carbon response to CMIP5 climate change projections. *Environ. Res. Lett.* 7:044008 10.1088/1748-9326/7/4/044008

[B6] Albert C. Bonn A. Burkhard B. Daube S. Dietrich K. Engels B. (2016a). Towards a national set of ecosystem service indicators: insights from Germany. *Ecol. Indic.* 61 38–48. 10.1016/j.ecolind.2015.08.050

[B7] Albert C. Galler C. Hermes J. Neuendorf F. von Haaren C. Lovett A. (2016b). Applying ecosystem services indicators in landscape planning and management: the ES-in-Planning framework. *Ecol. Indic.* 61 100–113. 10.1016/j.ecolind.2015.03.029

[B8] Anav A. Friedlingstein P. Kidston M. Bopp L. Ciais P. Cox P. (2013). Evaluating the land and ocean components of the global carbon cycle in the CMIP5 earth system models. *J. Clim.* 26 6801–6843. 10.1175/JCLI-D-12-00417.1

[B9] Angelstam P. Naumov V. Elbakidze M. Manton M. Priednieks J. Rendenieks Z. (2019). Wood production and biodiversity conservation are rival forestry objectives in Europe’s Baltic Sea Region. *Ecosphere* 9:e02119 10.1002/ecs2.2119

[B10] Annila E. (1969). Influence of temperature upon the development and voltinism of *Ips typographus* (L.) (Coleoptera, Scolytidae). *Ann. Zool. Fennici* 6 161–208. 10.1111/phen.12200 28979060 PMC5599993

[B11] Arslan A. N. Tanis C. M. Metsämäki S. Aurela M. Böttcher K. Linkosalmi M. (2017). Automated webcam monitoring of fractional snow cover in northern boreal conditions. *Geosciences* 7:55 10.3390/geosciences7030055

[B12] Barichivich J. Briffa K. R. Osborn T. J. Melvin T. M. Caesar J. (2012). Thermal growing season and timing of biospheric carbon uptake across the Northern Hemisphere. *Glob. Biogeochem. Cycles* 26:GB4015 10.1029/2012GB004312

[B13] Blennow K. (2012). Adaptation of forest management to climate change among private individual forest owners in Sweden. *For. Policy Econ.* 24 41–47. 10.1016/j.forpol.2011.04.005

[B14] Blomqvist M. Kosunen M. Starr M. Kantola T. Holopainen M. Lyytikäinen-Saarenmaa P. (2018). Modelling the predisposition of Norway spruce to *Ips typographus* L. infestation by means of environmental factors in southern Finland. *Eur. J. For. Res.* 137 675–691. 10.1007/s10342-018-1133-0

[B15] Böttcher K. Markkanen T. Thum T. Aalto T. Aurela M. Reick C. H. (2016). Evaluating biosphere model estimates of the start of the vegetation active season in boreal forests by satellite observations. *Remote Sens.* 8:580 10.3390/rs8070580

[B16] Brandt J. P. Flannigan M. D. Maynard D. G. Thompson I. D. Volney W. J. A. (2013). An introduction to Canada’s boreal zone: ecosystem processes, health, sustainability, and environmental issues. *Environ. Rev.* 21 207–226. 10.1139/er-2013-0040

[B17] Burkhard B. Kroll F. Müller F. Windhorst W. (2009). Landscapes’ capacities to provide ecosystem services – a concept for land-cover based assessments. *Landsc. Online* 15 1–22. 10.3097/LO.200915

[B18] Cadieux P. Boulanger Y. Cyr D. Taylor A. R. Price D. T. Tremblay J. A. (2019). Spatially explicit climate change projections for the recovery planning of threatened species: the Bicknell’s Thrush (*Catharus bicknelli*) as a case study. *Glob. Ecol. Conserv.* 17:e00530 10.1016/j.gecco.2019.e00530

[B19] Cajander A. K. (1949). Finnish forest types and their significance. *Acta For. Fenn.* 56 1–71. 10.1038/ng.3097 25282103 PMC4250049

[B20] CLC (2012). *CORINE Land Cover Dataset.* Available at: http://www.syke.fi/openinformation [accessed July 11 2018].

[B21] Costanza R. d’Arge R. De Groot R. Farber S. Grasso M. Hannon B. (1997). The value of the world’s ecosystem services and natural capital. *Nature* 387 253–260. 10.1126/sciadv.1601880 28435876 PMC5381958

[B22] Costanza R. De Groot R. Braat L. Kubiszewski I. Fioramonti L. Sutton P. (2017). Twenty years of ecosystem services: how far have we come and how far do we still need to go? *Ecosyst. Serv.* 28 1–16. 10.1016/j.ecoser.2017.09.008

[B23] Daily G. C. Matson P. (2008). Ecosystem services: from theory to implementation. *Proc. Natl. Acad. Sci. U.S.A.* 105 9455–9456. 10.1073/pnas.0804960105 18621697 PMC2474530

[B24] European Commission (2011). *Our Life Insurance, Our Natural Capital: an EU Biodiversity Strategy to 2020.* Brussels: European Commission 244.

[B25] European Commission (2018). *European Commission Mapping and Assessment of Ecosystems and their Services (MAES), 5th Report.* Available at: https://biodiversity.europa.eu/maes

[B26] Eyvindson K. Repo A. Mönkkönen M. (2018). Mitigating forest biodiversity and ecosystem service losses in the era of bio-based economy. *For. Policy Econ.* 92 119–127. 10.1016/j.forpol.2018.04.009

[B27] Fält-Nardmann J. J. J. Tikkanen O. P. Ruohomäki K. Lutz-Florian O. Leinonen R. Pöyry J. (2018). The recent northward expansion of *Lymantria monacha* in relation to realised changes in temperatures of different seasons. *For. Ecol. Manage.* 427 96–105. 10.1016/j.foreco.2018.05.053

[B28] FAO (2018). *The State of the World’s Forests 2018 - Forest Pathways to Sustainable Development.* Rome: FAO.

[B29] Farquhar G. D. Von Caemmerer S. Berry J. A. (1980). A biochemical model of photosynthetic carbon dioxide assimilation in leaves of 3-carbon pathway species. *Planta* 149 78–90. 10.1007/BF00386231 24306196

[B30] Finnish Climate Change Panel (2018). *Considerations Regarding Long-Term Emission Reduction Targets (in Finnish, Ilmastopaneelin Näkemykset Pitkän Aikavälin Päästövähennystavoitteen Asettamisessa Huomioon Otettavista Seikoista).* Available at: http://www.ilmastopaneeli.fi/fi/in-english/

[B31] Finnish Ecosystem Service Indicators (2015). Available at: www.biodiversity.fi/ecosystemservices Updated 16.1.2015 [accessed September 18 2018].

[B32] Forsius M. Akujärvi A. Mattsson T. Holmberg M. Punttila P. Posch M. (2016). Modelling impacts of forest bioenergy use on ecosystem sustainability: Lammi LTER region, southern Finland. *Ecol. Indic.* 65 66–75. 10.1016/j.ecolind.2015.11.032

[B33] Foster J. R. Townsend P. A. Mladenoff D. J. (2013). Mapping asynchrony between gypsy moth egg-hatch and forest leaf-out: putting the phenological window hypothesis in a spatial context. *For. Ecol. Manage.* 287 67–76. 10.1016/j.foreco.2012.09.006

[B34] Gao Y. Markkanen T. Thum T. Aurela M. Lohila A. Mammarella I. (2016). Assessing various drought indicators in representing summer drought in boreal forests in Finland. *Hydrol. Earth Syst. Sci.* 20 175–191. 10.5194/hess-20-175-2016

[B35] Gauthier S. Bernier P. Kuuluvainen T. Shvidenko A. Z. Schepaschenko D. G. (2015). Boreal forest health and global change. *Science* 349 819–822. 10.1126/science.aaa9092 26293953

[B36] Goll D. S. Brovkin V. Liski J. Raddatz T. Thum T. Todd-Brown K. E. O. (2015). Strong dependence of CO2 emissions from anthropogenic land cover change on initial land cover and soil carbon parametrization. *Glob. Biogeochem. Cycles* 29 1511–1523. 10.1002/2014GB004988

[B37] Hagemann S. Stacke T. (2015). Impact of the soil hydrology scheme on simulated soil moisture memory. *Clim. Dyn.* 44 1731–1750. 10.1007/s00382-014-2221-6

[B38] Haines-Young R. Potschin M. (2018). *Common International Classification of Ecosystem Services (CICES) V5.1 and Guidance on the Application of the Revised Structure.* Available at: http://www.cices.eu

[B39] Hedwall P. O. Brunet J. (2016). Trait variations of ground flora species disentangle the effects of global change and altered land-use in Swedish forests during 20 years. *Glob. Change Biol.* 22 4038–4047. 10.1111/gcb.13329 27111238

[B40] Heikkinen R. K. Pöyry J. Virkkala R. Bocedi G. Kuussaari M. Schweiger O. (2015). Modelling potential success of conservation translocations of a specialist grassland butterfly. *Biol. Conserv.* 192 200–206. 10.1016/j.biocon.2015.09.028

[B41] Hof R. H. Löfroth T. Rudolphi J. Work T. Hjälten J. (2018). Simulating long-term effects of bioenergy extraction on dead wood availability at a landscape scale in Sweden. *Forests* 9:457 10.3390/f9080457

[B42] Hökkä H. Uusitalo J. Lindeman H. Ala-Ilomäki J. (2016). Performance of weather parameters in predicting growing season water table depth variations on drained forested peatlands – a case study from southern Finland. *Silva Fenn.* 50:1687 10.14214/sf.1687

[B43] IPCC (2013). *Climate Change 2013: The Physical Science Basis. Contribution of Working Group I to the Fifth Assessment Report of the Intergovernmental Panel on Climate Change* eds Stocker T. F. Qin D. Plattner G.-K. Tignor M. Allen S. K. Boschung J. (Cambridge: Cambridge University Press) 1535.

[B44] Jeganathan C. Dash J. Atkinson P. M. (2014). Remotely sensed trends in the phenology of northern high latitude terrestrial vegetation, controlling for land cover change and vegetation type. *Remote Sens. Environ.* 143 154–170. 10.1016/j.rse.2013.11.020

[B45] Kalliokoski T. Mäkelä A. Fronzek S. Minunno F. Peltoniemi M. (2018). Decomposing sources of uncertainty in climate change projections of boreal forest primary production. *Agric. For. Meteorol.* 262 192–205. 10.1016/j.agrformet.2018.06.030

[B46] Kattge J. Knorr W. Raddatz T. Wirth C. (2009). Quantifying photosynthetic capacity and its relationship to leaf nitrogen content for global-scale terrestrial biosphere models. *Glob. Change Biol.* 15 976–991. 10.1093/jxb/44.5.907

[B47] Knorr W. (2000). Annual and interannual CO2 exchanges of the terrestrial biosphere: process-based simulations and uncertainties. *Glob. Ecol. Biogeogr.* 9 225–252. 10.1046/j.1365-2699.2000.00159.x

[B48] Knorr W. Heimann M. (1995). Impact of drought stress and other factors on seasonal land biosphere CO2 exchange studied through an atmospheric tracer transport model. *Tellus Ser. B* 47 471–489. 10.3402/tellusb.v47i4.16062

[B49] Kokkila M. (2013). *Ilmastonmuutoksen Vaikutus Puunkorjuun Talvikauden Korjuuoloihin Hienojakoisella Kivennäismaalla. Metsätieteen Aikakauskirja 1/2013:5–18 (In Finnish).* Available at: http://www.metla.fi/aikakauskirja/abs/fa13/fa131005.htm 10.14214/ma.6028

[B50] Kolari P. Lappalainen H. K. Hänninen H. Hari P. (2007). Relationship between temperature and the seasonal course of photosynthesis in Scots pine at northern timberline and in southern boreal zone. *Tellus* 59B 542–552. 10.1111/j.1600-0889.2007.00262.x

[B51] Kopperoinen L. Itkonen P. Niemelä J. (2014). Using expert knowledge in combining green infrastructure and ecosystem services in land use planning: an insight into a new place-based methodology. *Landsc. Ecol.* 29 1361–1375. 10.1007/s10980-014-0014-2

[B52] Kurz W. A. Shaw C. H. Boisvenue C. Stinson G. Metsaranta J. Leckie D. (2013). Carbon in Canada’s boreal forest – A synthesis. *Environ. Rev.* 21 260–292. 10.1139/er-2013-0041

[B53] Kuuluvainen T. Gauthier S. (2018). Young and old forest in the boreal: critical stages of ecosystem dynamics and management under global change. *For. Ecosyst.* 5:26 10.1186/s40663-018-0142-2

[B54] Laakkonen A. Zimmerer R. Kähkönen T. Hujala T. Takala T. Tikkanen J. (2018). Forest owner’s attitudes toward pro-climate and climate-responsive forest management. *For. Policy Econ.* 87 1–10. 10.1016/j.forpol.2017.11.001

[B55] Lai T. Y. Salminen J. Jäppinen J. P. Koljonen S. Mononen L. Nieminen E. (2018). Bridging the gap between ecosystem service indicators and ecosystem accounting in Finland. *Ecol. Modell.* 377 51–65. 10.1016/j.ecolmodel.2018.03.006

[B56] Lange H. Okland B. Krokene P. (2006). Thresholds in the life cycle of the spruce bark beetle under climate change. *Int. Complex Syst.* 1648 1–10.

[B57] Lankia T. Kopperoinen L. Pouta E. Neuvonen M. (2015). Valuing recreational ecosystem service flow in Finland. *J. Outdoor Recreat. Tour.* 10 14–28. 10.1016/j.jort.2015.04.006

[B58] Lappalainen H. K. Kerminen V. M. Petäjä T. Kurten T. Baklanov A. Shvidenko A. (2016). Pan-Eurasian Experiment (PEEX): towards a holistic understanding of the feedbacks and interactions in the land –atmosphere –ocean –society continuum in the northern Eurasian region. *Atmos. Chem. Phys.* 16 14461–14461. 10.5194/acp-16-14421-2016

[B59] Lindenmayer D. Franklin J. Lõhmus A. Baker S. Bauhus J. Beese W. (2012). A major shift to the retention approach for forestry can help resolve some global forest sustainability issues. *Conserv. Lett.* 5 421–431. 10.1111/j.1755-263X.2012.00257.x

[B60] Liski J. Westman C. J. (1995). Density of organic carbon in soil at coniferous forest sites in southern Finland. *Biogeochemistry* 29 183–197. 10.1007/BF02186047

[B61] Liu Y. Y. Van Dijk A. I. J. M. De Jeu R. A. M. Canadell J. G. McCabe M. F. Evans J. P. (2015). Recent reversal in loss of global terrestrial biomass. *Nat. Clim. Change* 5 470–474. 10.1038/NCLIMATE258

[B62] Ludwig G. X. Alatalo R. V. Helle P. Lindén H. Lindström J. Siitari H. (2006). Short-and long-term population dynamical consequences of asymmetric climate change in black grouse. *Proc. R. Soc. B* 273 2009–2016. 10.1098/rspb.2006.3538 16846907 PMC1635476

[B63] LUKE (2018a). *Forest Resources Open Data File Service.* Available at: http://kartta.luke.fi/index-en.html

[B64] LUKE (2018b). *Forest Resources by Region.* Available at: http://stat.luke.fi/en/forest-resources

[B65] Luyssaert S. Marie G. Valade A. Chen Y. Y. Djomo S. N. Ryder J. (2018). Trade-offs in using European forests to meet climate objectives. *Nature* 562 259–262. 10.1038/s41586-018-0577-1 30305744 PMC6277009

[B66] Maes J. Hauck J. Paracchini M. L. Ratamäki O. Hutchins M. Termansen M. (2013). Mainstreaming ecosystem services into EU policy. *Curr. Opin. Environ. Sustain.* 5 128–134. 10.1016/j.cosust.2013.01.002

[B67] Mäkelä A. Hari P. Berninger F. Hänninen H. Nikinmaa E. (2004). Acclimation of photosynthetic capacity in Scots pine to the annual cycle temperature. *Tree Physiol.* 24 369–376. 10.1093/treephys/24.4.369 14757576

[B68] Mäkelä A. Pulkkinen M. Mäkinen H. (2016). Bridging empirical and carbon-balance based forest site productivity – Significance of below-ground allocation. *For. Ecol. Manage.* 372 64–77. 10.1016/j.foreco.2016.03.059

[B69] Mäkisara K. Katila M. Peräsaari J. Tomppo E. (2016). The multi-source national forest inventory of Finland – methods and results 2013. *Nat. Resour. Bioeconomy Stud.* 10:215.

[B70] McKenney D. W. Pedlar J. H. Papadopol P. Hutchinson M. F. (2006). The development of 1901 – 2000 historical monthly climate models for Canada and the United States. *Agric. For. Meteorol.* 138 69–81. 10.1016/j.agrformet.2006.03.012

[B71] MEA (2005). *Millennium Ecosystem Assessment: Living Beyond Our Means – Natural Assets and Human Well-Being.* Washington DC: World Resources Institute.

[B72] Meehl G. A. Goddard L. Murphy J. Stouffer R. J. Boer G. Danabasoglu G. (2009). Decadal prediction: can it be skillful? *Bull. Am. Meteor. Soc.* 90 1467–1485. 10.1175/2009BAMS2778.1

[B73] MEK (2010). *Kansainvälinen Luontomatkailututkimus. Matkailun Edistämiskeskus (Visit Finland) 169 2010 (In Finnish).* Available at: http://www.visitfinland.fi/studies/kansainvalinen-luontomatkailututkimus-2010/

[B74] Metadata (2018a). *Metadata for JSBACH Simulations*. Available at: http://metatieto.ymparisto.fi:8080/geoportal/catalog/search/resource/details.page?uuid=%7B7AC01098-01EC-411E-81F3-7A7DB7111760%7D

[B75] Metadata (2018b). *Metadata for PREBAS Simulations.* Available at: http://metatieto.ymparisto.fi:8080/geoportal/catalog/search/resource/details.page?uuid=%7B186E1CEB-9361-4597-A3BA-EBE236D461AC%7D

[B76] Mikkonen S. Laine M. Mäkelä H. M. Gregow H. Tuomenvirta H. Lahtinen M. (2015). Trends in the average temperature in Finland, 1847–2013. *Stoch. Environ. Res. Risk Assess.* 29 1521–1529. 10.1007/s00477-014-0992-2

[B77] Minunno F. Peltoniemi M. Härkönen S. Kalliokoski T. Makinen H. Mäkelä A. (2019). Bayesian calibration of a carbon balance model PREBAS using data from permanent growth experiments and national forest inventory. *For. Ecol. Manage.* 440 208–257. 10.1016/j.foreco.2019.02.041

[B78] Minunno F. Peltoniemi M. Launiainen S. Aurela M. Lindroth A. Lohila A. (2016). Calibration and validation of a semi-empirical flux ecosystem model for coniferous forests in the Boreal region. *Ecol. Modell.* 341 37–52. 10.1016/j.ecolmodel.2016.09.020

[B79] MONIMET (2017). *LIFE12 ENV/FI/000409 Climate Change Indicators and Vulnerability of Boreal Zone Applying Innovative Observation and Modelling Techniques.* Available at: http://monimet.fmi.fi/project/deliverables/Action_E1/MONIMET_LIFE_ENV_FIN_000409_FINAL_Report.pdf

[B80] Mönkkönen M. Juutinen A. Mazziotta A. Miettinen K. Podkopaev D. Reunanen P. (2014). Spatially dynamic forest management to sustain biodiversity and economic returns. *J. Environ. Manag.* 134 80–89. 10.1016/j.jenvman.2013.12.021 24463852

[B81] Mononen L. Auvinen A. P. Ahokumpu A. L. Rönkä M. Aarras N. Tolvanen H. (2016). National ecosystem service indicators: measures of social-ecological sustainability. *Ecol. Indic.* 61 27–37. 10.1016/j.ecolind.2015.03.041

[B82] Montoro Girona M. Morin H. Lussier J.-M. Walsh D. (2016). Radial growth response of black spruce stands ten years after experimental shelterwoods and seed-tree cuttings in boreal forest. *Forests* 7:240 10.3390/f7100240

[B83] Montoro Girona M. Morin H. H. Lussier J.-M. J. Thiffault N. N. (2018). Conifer regeneration after experimental shelterwood and seed-tree treatments in boreal forests: finding silvicultural alternatives. *Front. Plant Sci.* 9:1145. 10.3389/fpls.2018.01145 30174675 PMC6108379

[B84] Montoro Girona M. Rossi S. Lussier J.-M. Walsh D. Morin H. (2017). Understanding tree growth responses after partial cuttings: a new approach. *PLoS One* 12:e0172653. 10.1371/journal.pone.0172653 28222200 PMC5319695

[B85] Moss R. H. Edmonds J. A. Hibbard K. A. Manning M. R. Rose S. K. Van Vuuren D. P. (2010). The next generation of scenarios for climate change research and assessment. *Nature* 463 747–756. 10.1038/nature08823 20148028

[B86] Muukkonen P. Nevalainen S. Lindgren M. Peltoniemi M. (2015). Spatial occurrence of drought-associated damages in Finnish boreal forests: results from forest condition monitoring and GIS analysis. *Boreal Environ. Res.* 20 172–180.

[B87] Navarro L. Morin H. Bergeron Y. Girona M. M. (2018). Changes in spatiotemporal patterns of 20th century spruce budworm outbreaks in eastern Canadian boreal forests. *Front. Plant Sci.* 9:1905. 10.3389/fpls.2018.01905 30622551 PMC6308396

[B88] Neuvonen M. Sievänen T. (2011). “Ulkoilutilastot 2010 (Statistical tables of recreational activities 2010, in Finnish),” in *Luonnon virkistyskäyttö* 2010 Vol. 212 eds Sievänen T. Neuvonen M. (Dordrecht: Metlan työraportteja) 133–190.

[B89] Neuvonen M. Sievänen T. Fronzek S. Lahtinen I. Veijalainen N. Carter T. R. (2015). Vulnerability of cross-country skiing to climate change in Finland – An interactive mapping tool. *J. Outdoor Recreat. Tour.* 11 64–79. 10.1016/j.jort.2015.06.010

[B90] Official Statistics of Finland (2019). *OSF: Greenhouse Gases [e-Publication]. ISSN = 1797-6065. Land use, Land-use Change and Forestry, Preliminary Data 2017.* Helsinki: Statistics Finland.

[B91] Pan Y. Birdsey R. A. Fang J. Houghton R. Kauppi P. E. Kurz W. A. (2011). A large and persistent carbon sink in the world’s forests. *Science* 333 988–993. 10.1126/science.1201609 21764754

[B92] Peltoniemi M. Aurela M. Böttcher K. Kolari P. Loehr J. Hokkanen T. (2018a). Networked web-cameras monitor congruent seasonal development of birches with phenological field observations. *Agric. For. Meteorol.* 249 335–347. 10.1016/j.agrformet.2017.10.008

[B93] Peltoniemi M. Aurela M. Böttcher K. Kolari P. Loehr J. Karhu J. (2018b). Webcam network and image database for studies of phenological changes of vegetation and snow cover in Finland, image time series from 2014 to 2016. *Earth Syst. Sci. Data* 10 173–184. 10.5194/essd-10-173-2018

[B94] Peltoniemi M. Markkanen T. Härkönen S. Muukkonen P. Thum T. Aalto T. (2015a). Consistent estimates of gross primary production of Finnish forests — comparison of estimates of two process models. *Boreal Environ. Res.* 20 196–212.

[B95] Peltoniemi M. Pulkkinen M. Aurela M. Pumpanen J. Kolari P. Mäkelä A. (2015b). A semi-empirical model of boreal forest gross primary production, evapotranspiration, and soil water – calibration and sensitivity analysis. *Boreal Environ. Res.* 20 151–171.

[B96] Piao S. Wang X. Ciais P. Zhu B. Wang T. Liu J. (2011). Changes in satellite-derived vegetation growth trend in temperate and boreal Eurasia from 1982 to 2006. *Glob. Change Biol.* 17 3228–3239. 10.1111/j.1365-2486.2011.02419.x

[B97] Pohjola J. Laturi J. Lintunen J. Uusivuori J. (2018). Immediate and long-run impacts of a forest carbon policy – A market-level assessment with heterogeneous forest owners. *J. For. Econ.* 32 94–105. 10.1016/j.jfe.2018.03.001

[B98] Potschin M. Haines-Young R. (2011). Ecosystem services: exploring a geographical perspective. *Prog. Phys. Geogr.* 35 575–594. 10.1177/0309133311423172

[B99] Potschin M. Haines-Young R. (2013). Landscapes, sustainability and the place-based analysis of ecosystem services. *Landsc. Ecol.* 28 1053–1065. 10.1007/s10980-012-9756-x

[B100] Pöyry J. Leinonen R. Söderman G. Nieminen M. Heikkinen R. K. Carter T. (2011). Climate-induced increase of moth multivoltinism in boreal regions. *Glob. Ecol. Biogeogr.* 20 289–298. 10.1111/j.1466-8238.2010.00597.x

[B101] Price D. T. Alfaro R. I. Brown K. J. Flannigan M. D. Fleming R. A. Hogg E. H. (2013). Anticipating the consequences of climate change for Canada’s boreal forest ecosystems. *Environ. Rev.* 21 322–365. 10.1139/er-2013-0042

[B102] Pukkala T. (2018). Effect of species composition on ecosystem services in European boreal forest. *J. For. Res.* 29 261–272. 10.1007/s11676-017-0576-3

[B103] Pureswaran D. S. De Grandpré L. Paré D. Taylor A. Barrette M. Morin H. (2015). Climate induced changes in host tree–insect phenology may drive ecological state shift in boreal forests. *Ecology* 96 1480–1491. 10.1890/13-2366.1

[B104] Raddatz T. Z. Reick C. H. Knorr W. Kattge J. Roeckner E. Schnur R. (2007). Will the tropical land biosphere dominate the climate-cargon dynamic feedback during the twenty-first century? *Clim. Dyn.* 29 565–574. 10.1007/s00382-007-0247-8

[B105] Räisänen J. Räty O. (2013). Projections of daily mean temperature variability in the future: cross-validation tests with ENSEMBLES regional climate simulations. *Clim. Dyn.* 41 1553–1568. 10.1007/s00382-012-1515-9

[B106] Räty O. Räisänen J. Ylhäisi J. S. (2014). Evaluation of delta change and bias correction methods for future daily precipitation: intermodal cross-validation using ENSEMBLES simulations. *Clim. Dyn.* 42 2287–2303. 10.1007/s00382-014-2130-8

[B107] Reick C. H. Raddatz T. Brovkin V. Gayler V. (2013). Representation of natural and anthropogenic land cover change in MPI-ESM. *J. Adv. Model. Earth Syst.* 5 459–482. 10.1002/jame.20022

[B108] Rockström J. Gaffney O. Rogelj J. Meinshausen M. Nakicenovic N. Schellnhuber H. J. (2017). A roadmap for rapid decarbonization. *Science* 355 1269–1271. 10.1126/science.aah3443 28336628

[B109] Rogelj J. Popp A. Calvin K. V. Luderer G. Emmerling J. Gernaat D. (2018). Scenarios towards limiting global mean temperature increase below 1.5 °C. *Nat. Clim. Change* 8 325–332. 10.1038/s41558-018-0091-3

[B110] Ruosteenoja K. Jylhä K. Kämäräinen M. (2016). Climate projections for Finland under the RCP forcing scenarios. *Geophysica* 51 17–50.

[B111] Saastamoinen O. Matero J. Horne P. Kniivilä M. Haltia E. Vaara M. (2014). *Classification of Boreal Forest Ecosystem Goods and Services in Finland. Publications of the University of Eastern Finland. Reports and Studies in Forestry and Natural Sciences Number 11.* Kuopio: University of Eastern Finland.

[B112] Schaphoff S. Reyer C. P. O. Schepaschenko D. Gerten D. Shvidenko A. (2016). Tamm Review: observed and projected climate change impacts on Russia’s forests and its carbon balance. *For. Ecol. Manage.* 361 432–444. 10.1016/j.foreco.2015.11.043

[B113] Schröter M. Albert C. Marques A. Tobon W. Lavorel S. Maes J. (2016). National ecosystem assessments in Europe: a review. *Bioscience* 66 813–828. 10.1093/biosci/biw101 28533561 PMC5421311

[B141] Schulze E.-D. Kelliher F. M. Körner C. Lloyd J. Leuning R. (1994). Relationships among maximum stomatal conductance, ecosystem surface conductance, carbon assimilation rate, and plant nitrogen nutrition: a global ecology scaling exercise. *Annu. Rev. Ecol. Syst.* 25 629–660. 10.1146/annurev.es.25.110194.003213

[B114] Sellers P. J. (1985). Canopy reflectance, photosynthesis and transpiration. *Int. J. Remote Sens.* 6 1335–1372. 10.1080/01431168508948283

[B115] Shao P. Zeng X. Sakaguchi K. Monson R. K. Zeng X. (2013). Terrestrial carbon cycle: climate relations in eight CMIP5 earth system models. *J. Clim.* 26 8744–8764. 10.1175/JCLI-D-12-00831.1

[B116] Shvidenko A. Barber C. V. Persson R. Gonzalez P. Hassan R. Lakyda P. (2005). *Forest and Woodland Systems. Chapter 21 in Current State & Trends Assessment of the Millennium Assessment.* Available at: https://www.millenniumassessment.org/documents/document.290.aspx.pdf

[B117] Skogsdata (2018). *Forest Statistics 2018 (In Swedish with English Captions). Official Statistics of Sweden.* Umeå: Swedish University of Agricultural Sciences.

[B118] Soimakallio S. Saikku L. Valsta L. Pingoud K. (2016). Climate change mitigation challenge for wood utilization – the case of Finland. *Environ. Sci. Technol.* 50 5127–5134. 10.1021/acs.est.6b00122 27074531

[B119] Sousa-Silva R. Verbist B. Lomba A. Valent P. Suškeviès M. Picard O. (2018). Adapting forest management to climate change in Europe: linking perceptions to adaptive responses. *For. Policy Econ.* 90 22–30. 10.1016/j.forpol.2018.01.004

[B120] Suvinen A. (2006). A GIS-based simulation model for terrain tractability. *J. Terramechanics* 43 427–449. 10.1016/j.jterra.2005.05.002

[B121] Sved J. Koistinen A. (eds) (2015). *Metsänhoidon Suositukset Kannattavaan Metsätalouteen, Työopas. In Finnish (Best Practices for Profitable Forest Management).* Available at: https://www.metsanhoitosuositukset.fi/wp-content/uploads/2016/09/Metsanhoidon_suositukset_kannattavaan_metsatalouteen_Tapio_2015_B.pdf [accessed July 11 2018].

[B122] SYKE (2018). *Vegetation Phenology 2001-2017. Maps of the Start of the Vegetation Active Period in Finland.* Available at: http://syke.maps.arcgis.com/apps/webappviewer/index.html?id=a446e987496b4d8794b307e882da718a [accessed October 5 2018].

[B123] Taylor K. E. Stouffer R. J. Meehl G. A. (2012). An Overview of CMIP5 and the experiment design. *Bull. Am. Meteor. Soc.* 93 485–498. 10.1175/BAMS-D-11-00094.1

[B124] Thomas C. D. Anderson B. J. Moilanen A. Eigenbrod F. Heinemeyer A. Quaife T. (2013). Reconciling biodiversity and carbon conservation. *Ecol. Lett.* 16 39–47. 10.1111/ele.12054 23279784

[B125] Tomppo E. Katila M. Mäkisara K. Peräsaari J. (2014). *The Multi-Source National Forest Inventory of Finland – Methods and Results 2011.* Available at: http://urn.fi/URN:ISBN:978-951-40-2516-7

[B126] Tremblay J. A. Boulanger Y. Cyr D. Taylor A. R. Price D. T. St-Laurent M.-H. (2018). Harvesting interacts with climate change to affect future habitat quality of a focal species in eastern Canada’s boreal forest. *PLoS One* 13:e0191645. 10.1371/journal.pone.0191645 29414989 PMC5802891

[B127] Tuomi M. Thum T. Järvinen H. Fronzek S. Berg B. Harmo M. (2009). Leaf litter decomposition – Estimates of global variability based on Yasso07 model. *Ecol. Modell.* 220 3362–3371. 10.1016/j.ecolmodel.2009.05.016

[B128] Vaahtera E. Aarne M. Ihalainen A. Mäki-Simola E. Peltola A. Torvelainen J. (2018). *Metsätilastot – Finnish Forest Statistics (In Finnish and English). Finnish Natural Resources Institute LUKE.* Available at: https://stat.luke.fi/sites/default/files/suomen_metsatilastot_2018_verkko.pdf

[B129] Valentine H. T. Mäkelä A. (2005). Bridging process-based and empirical approaches to modeling tree growth. *Tree Physiol.* 25 769–779. 10.1093/treephys/25.7.769 15870047

[B130] Van Oudenhoven A. P. E. Aukes E. Bontje L. E. Vikolainen V. van Bodegom P. M. Slinger J. H. (2018). ‘Mind the Gap’ between ecosystem classification and strategic decision making. *Ecosyst. Serv.* 33 77–88. 10.1016/j.ecoser.2018.09.003

[B131] Van Vuuren D. P. Edmonds J. Kainuma M. Riahi K. Thomson A. Hibbard K. (2011). The representative concentration pathways: an overview. *Clim. Change* 109 5–13. 10.1007/s10584-011-0148-z

[B132] Vanhala P. Bergström I. Haaspuro T. Kortelainen P. Holmberg M. Forsius M. (2016). Boreal forests can have a remarkable role in reducing greenhouse gas emissions locally: land use-related and anthropogenic greenhouse gas emissions and sinks at the municipal level. *Sci. Total Environ.* 557–558 51–57. 10.1016/j.scitotenv.2016.03.040 26994793

[B133] Vauhkonen J. Ruotsalainen R. (2017). Assessing the provisioning potential of ecosystem services in a Scandinavian boreal forest: suitability and tradeoff analyses on grid-based wall-to-wall forest inventory data. *For. Ecol. Manage.* 389 272–284. 10.1016/j.foreco.2016.12.005

[B134] Vihervaara P. Kumpula T. Tanskanen A. Burkhard B. (2010). Ecosystem services – A tool for sustainable management of human-environment systems. Case study Finnish Forest Lapland. *Ecol. Complex.* 7 410–420. 10.1016/j.ecocom.2009.12.002

[B135] Virkkala R. (2016). Long-term decline of southern boreal forest birds: consequence of habitat alteration or climate change? *Biodivers. Conserv.* 25 151–167. 10.1007/s10531-015-1043-0

[B136] Virkkala R. Heikkinen R. K. Fronzek S. Kujala H. Leikola N. (2013). Does the protected area network preserve bird species of conservation concern in a rapidly changing climate? *Biodivers. Conserv.* 22 459–482. 10.1007/s10531-012-0423-y

[B137] Wegge P. Rolstad J. (2017). Climate change and bird reproduction: warmer springs benefit breeding success in boreal forest grouse. *Proc. R. Soc. B* 284:20171528. 10.1098/rspb.2017.1528 29118133 PMC5698643

[B138] Wieder W. R. Cleveland C. C. Smith W. K. Todd-Brown K. (2015). Future productivity and carbon storage limited by terrestrial nutrient availability. *Nat. Geosci.* 8 441–444. 10.1038/ngeo2413 22924405

[B139] Wullschleger S. D. (1993). Biochemical limitations to carbon assimilation in C3 plants – A retrospective analysis of the A/Ci curves from 109 species. *J. Exp. Bot.* 44 907–920. 10.1093/jxb/44.5.907

[B140] Zang C. Helm R. Sparks T. H. Menzel A. (2015). Forecasting bark beetle early flight activity with plant phenology. *Clim. Res.* 66 161–170. 10.3354/cr01346

