# Supplementary material for: Ecosystem Services Related to Carbon Cycling – Modeling Present and Future Impacts in Boreal Forests
Source: Front Plant Sci. 2019 Mar 26;10:343. doi: 10.3389/fpls.2019.00343 (PMC6443878; doi:10.3389/fpls.2019.00343)
Supplement: Supplementary file 1 [file Table_1.pdf]

Supplementary Material

Ecosystem services related to carbon cycling – modeling present and future impacts in boreal forests

Maria Holmberg*, Tuula Aalto, Anu Akujärvi, Ali Nadir Arslan, Irina Bergström, Kristin Böttcher, Ismo Lahtinen, Annikki Mäkelä, Tiina Markkanen, Francesco Minunno, Mikko Peltoniemi, Katri Rankinen, Petteri Vihervaara, Martin Forsius.

Frontiers in Plant Science. doi: 10.3389/fpls.2019.00343

*** Correspondence:** Corresponding Author: maria.holmberg@ymparisto.fi

1. Framework to assess carbon related ES in boreal forests

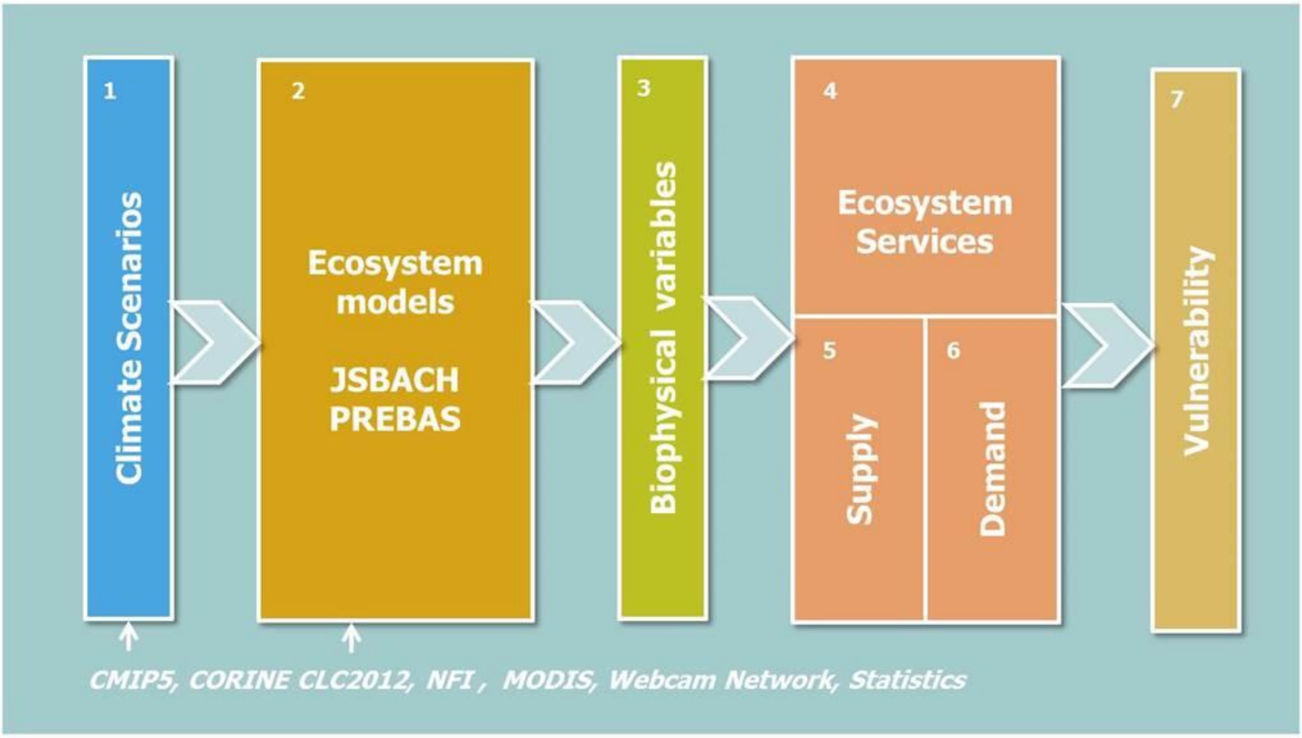

Supplementary Figure 1. Framework to assess carbon related ecosystem services (ES) in boreal forests. Climate change scenarios (1) are input to ecosystem models (2) to project future values of biophysical variables (3), which are interpreted as ES (4) and estimates of future supply (5) of ES can be made. As estimates of future demand (6) of ES are not available in most cases, we assessed vulnerability (7) in relation to changes in supply. In the assessment we used input information derived from research projects and infrastructures such as CMIP5 (Meehl et al., 2009, Taylor et al., 2012), CORINE (CLC, 2012), satellite observations (Böttcher et al., 2016). Our results were also supported by the continuous monitoring of the webcam network (Arslan et al., 2017, Peltoniemi et al., 2018a). Furthermore statistical data from Official Statistics of Finland (2019) and LUKE (2018a, 2018b) were used.

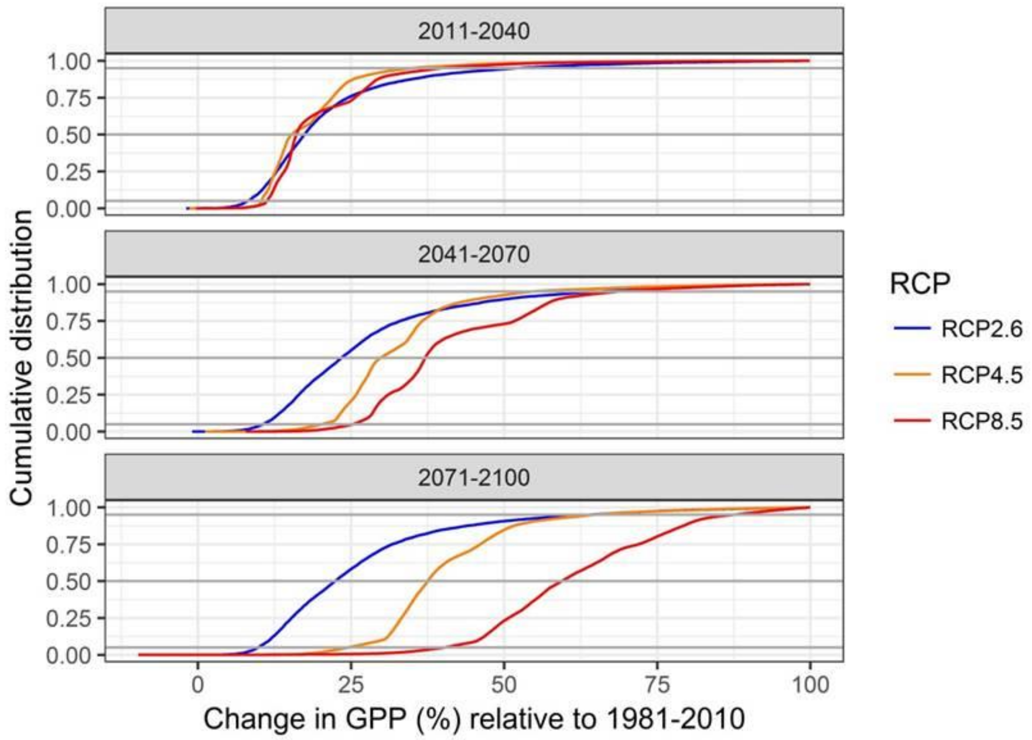

Supplementary Figure 2. Empirical cumulative distribution functions of change in GPP (%) relative to 1981–2010 by level of climate forcing (RP2.6, RCP4.5, RCP8.5) in each time period (2011–2040, 2041–2070, 2071–2099) as simulated by JSBACH and PREBAS for all of Finland. Horizontal grey lines indicate percentiles (5th, median, 95th). The percentile values are given in Supplementary Table 1.

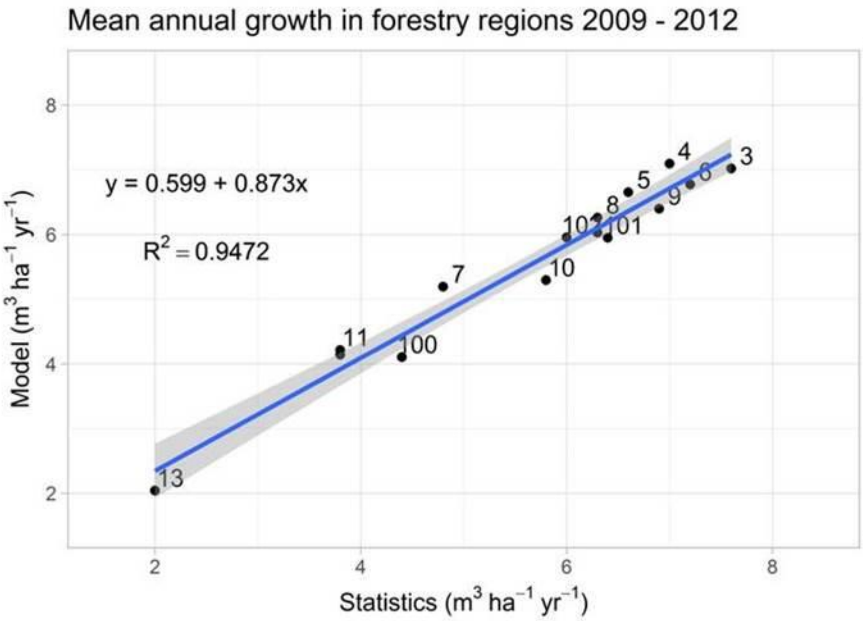

Code	Forestry region
100	Ahvenanmaa
101	Etelärannikko
102	Pohjanmaa
2	Lounais-Suomi
3	Häme-Uusimaa
4	Kaakkois-Suomi
5	Pirkanmaa
6	Etelä-Savo
7	Etelä-Keski-Pohjanmaa
8	Keski-Suomi
9	Pohjois-Savo
10	Pohjois-Karjala
11	Kainuu
12	Pohjois-Pohjanmaa
13	Lappi

Supplementary Figure 3. Mean annual growth in forestry regions as observed 2009 – 2012 (LUKE 2018b) compared to PREBAS simulation results for the same period.

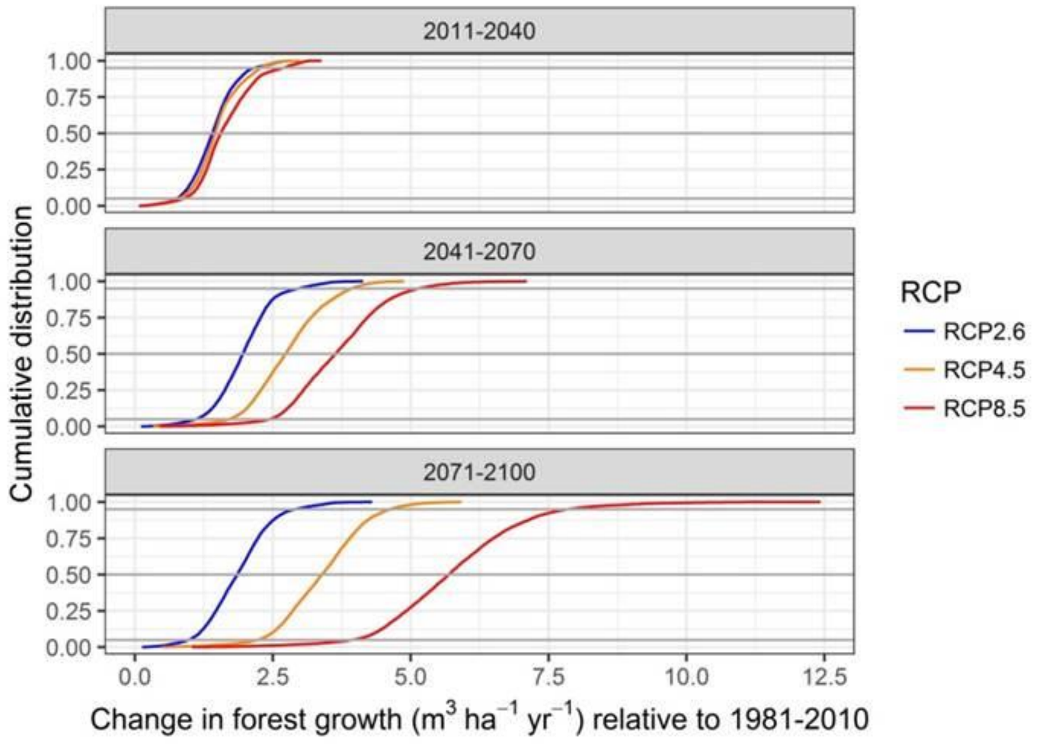

Supplementary Figure 4. Empirical cumulative distribution functions of change in forest growth ($m^3 ha^{-1} yr^{-1}$) relative to 1981–2010 by level of climate forcing (RP2.6, RCP4.5, RCP8.5) in each time period (2011–2040, 2041–2070, 2071–2099) as simulated by PREBAS for all of Finland. Horizontal grey lines indicate percentiles (5th, median, 95th). The percentile values are given in Supplementary Table 1.

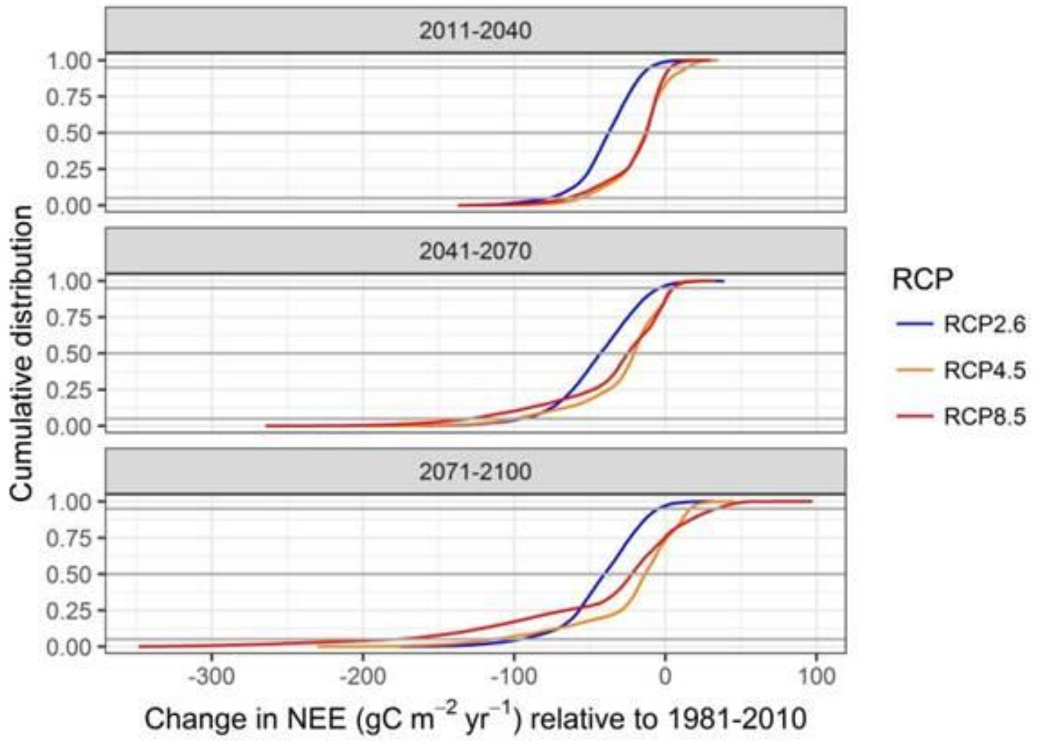

Supplementary Figure 5. Empirical cumulative distribution functions of change in NEE ($gC\ m^{-2}\ yr^{-1}$) relative to 1981–2010 by level of climate forcing (RP2.6, RCP4.5, RCP8.5) in each time period (2011–2040, 2041–2070, 2071–2099) as simulated by JSBACH and PREBAS for all of Finland. Horizontal grey lines indicate percentiles (5th, median, 95th). The percentile values are given in Supplementary Table 1.

Webcam network

A novel webcam network was designed and implemented to provide detailed information on phenological dynamics in order to validate ecosystem models (Peltoniemi et al. 2018a). Season start and end date estimates were obtained from color analyses of webcam images. In order to estimate the phenological transition dates, color analyses with subsequent time series analyses were made for the images of each site (n=12) and year (1-3 years of images). At each site, the color analyses focused on image sub-regions covered by birch trees (*B. pendula* or *B. pubescens*). For each image and sub-region, average green (GCC) and red chromatic coordinates (RCC) were calculated from the image pixel data by

$$GCC = \frac{G}{R + G + B}$$

$$RCC = \frac{R}{R + G + B}$$

where R, G, and B refer to digital numbers of red, green and blue channels of image pixels, respectively. For each site and day, medians of GCC and RCC were calculated from all images taken during the daylight time. The developments of GCC and RCC time logically tracked the color changes of birch canopies over the seasons at all studied sites and years.

The time-series of GCC and RCC were analyzed in more detail for the spring and autumn transition dates, which could correspond to leaf budburst and fall, i.e. VAPstart and VAPend. Here, we used the estimates of SOS of Peltoniemi et al. (2018a) as the estimates of VAPstart. By definition, SOS had the steepest GCC increase in the spring. Similarly, we used the EOSr as the estimate of VAPend, which by definition, was the date when RCC peaked in the autumn. See Peltoniemi et al. (2018a) for details of the methodology, and Peltoniemi et al. (2018b) for the original image data. For a set of observation sites in Finland, the JSBACH simulated results of the start and end of the vegetation active period were compared to those of the webcam images.

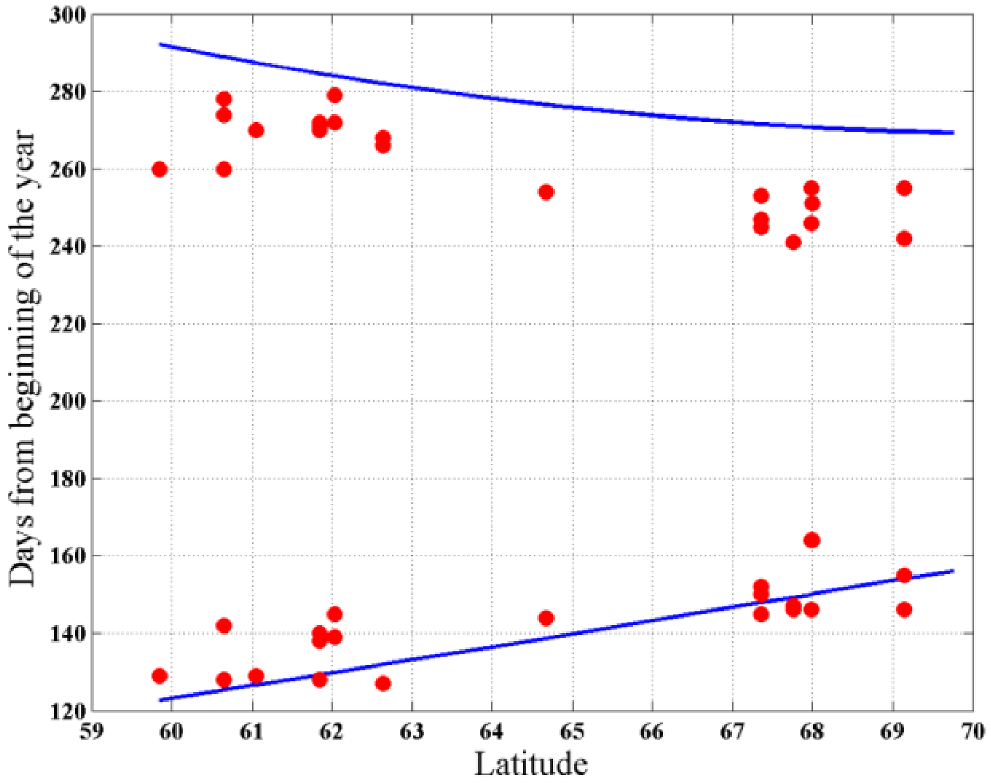

Supplementary Figure 6. Start and end of vegetation active period from Webcam results (red dots) and JSBACH simulated (blue lines) average values for a set of observation sites in Finland.

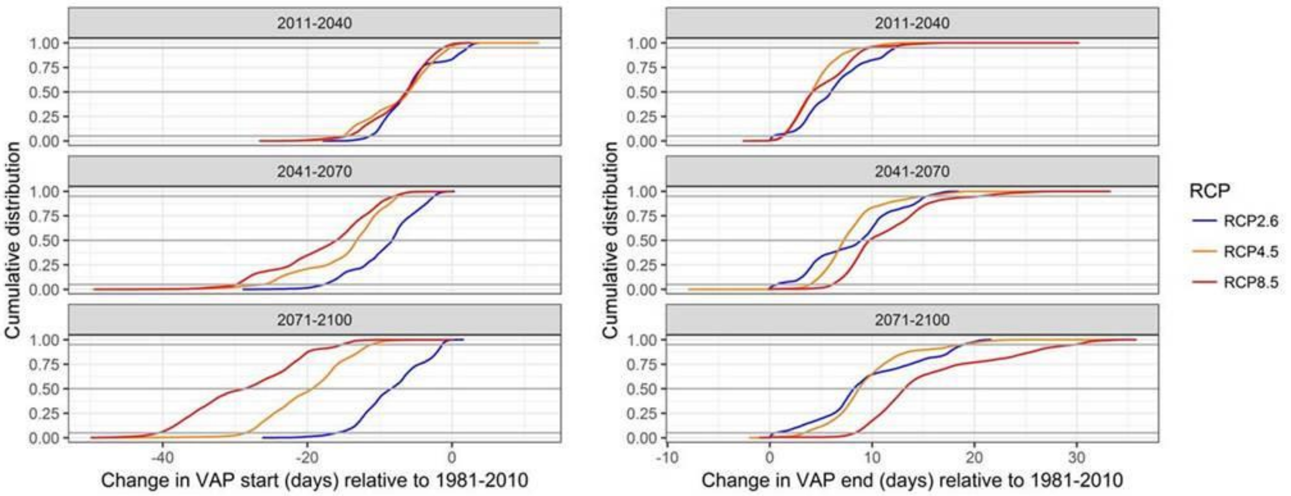

Supplementary Figure 7. Empirical cumulative distribution functions of change in VAPstart (days, left) and VAPend (days, right) relative to 1981–2010 by level of climate forcing (RP2.6, RCP4.5, RCP8.5) in each time period (2011–2040, 2041–2070, 2071–2099) as simulated by JSBACH and PREBAS for all of Finland. Horizontal grey lines indicate percentiles (5th, median, 95th). The percentile values are given in Supplementary Table 1.

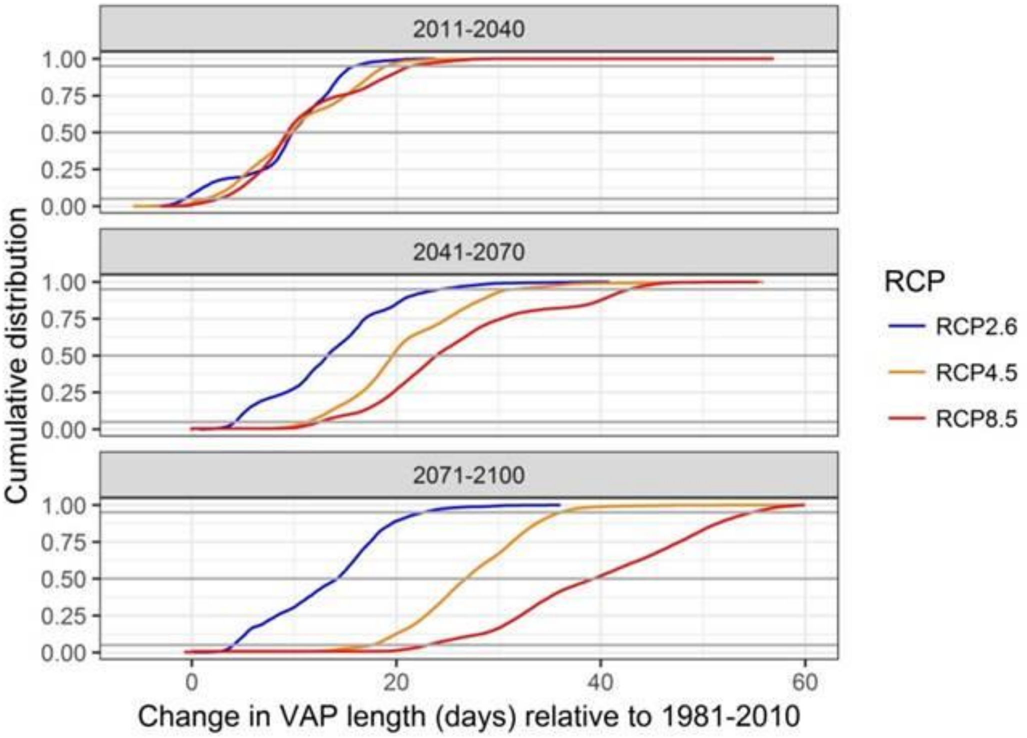

Supplementary Figure 8. Empirical cumulative distribution functions of change in VAPlength (days) relative to 1981–2010 by level of climate forcing (RP2.6, RCP4.5, RCP8.5) in each time period (2011–2040, 2041–2070, 2071–2099) as simulated by JSBACH and PREBAS for all of Finland. Horizontal grey lines indicate percentiles (5th, median, 95th). The percentile values are given in Supplementary Table 1.

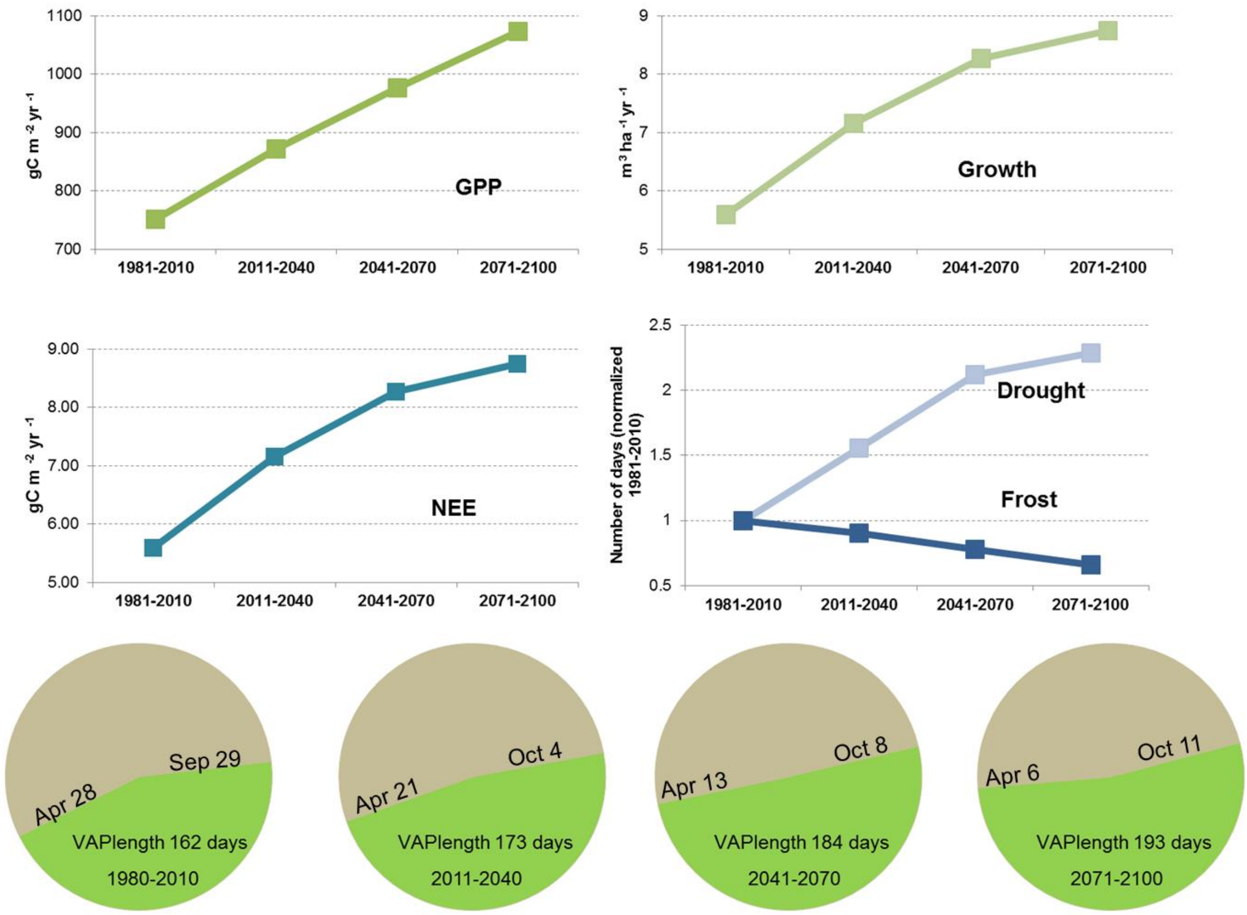

Supplementary Figure 9. Climate change impacts on biophysical proxies for key ES of boreal forests: Provisioning: GPP (gC m^{-2} yr^{-1}), forest growth (m^3 ha^{-1} yr^{-1}); Regulating: NEE (gC m^{-2} yr^{-1}), drought (normalized number of dry summer days), frost (normalized number or soil frost days); Regulating and cultural: Length of vegetation active period (number of days, with start and end dates indicated).

Supplementary Table 1. Summary of simulated results for biophysical variables for all of mainland Finland for the baseline period 1981–2010, and the future periods 2011–2040, 2041–2070, and 2071–2100. For the future periods, the change in variable values are calculated as differences: future value – baseline value. The results are obtained from simulations with PREBAS and JSBACH (except forest growth with PREBAS only) using future climate data from five climate models with RCP2.6, RPC4.5 and RCP8.5 (PREBAS), and RCP4.5 and RCP8.5 (JSBACH). The results are summarized as percentiles: 5th, median and 95th.

**Variable**	**1981–2010**			**2011–2040**			**2041–2070**			**2071–2100**		
	**5th**	**median**	**95th**	**5th**	**median**	**95th**	**5th**	**median**	**95th**	**5th**	**median**	**95th**
GPP (gC m^{-2} yr^{-1})	288	751	968	402	872	1136	509	977	1315	576	1073	1500
Change in GPP (%)	-	-	-	10	16	41	17	34	78	18	46	101
Forest growth (m^3 ha^{-1} yr^{-1})	0.9	5.6	6.9	2.0	7.2	8.5	2.9	8.3	10.2	3.4	8.7	12.4
Change in forest growth (m^3 ha^{-1} yr^{-1})	-	-	-	0.8	1.5	2.4	1.4	2.7	4.5	1.3	3.4	7.0
NEE (gC m^{-2} yr^{-1})	-213	-50	-19	-259	-62	-25	-288	-74	-28	-306	-66	-14
Change NEE (gC m^{-2} yr^{-1})	-	-	-	-63	-15	8	-111	-24	4	-137	-19	21
VAPlength (days)	127	162	197	137	173	208	149	184	220	156	193	229
Change in VAPlength (days)	-	-	-	1	10	20	10	20	40	11	30	52
VAPstart	Apr 5th	Apr 28th	May 20th	Mar 28th	Apr 21st	May 14th	Mar 18th	Apr 13th	May 6th	Mar 10th	Apr 6th	May 1st
Change in VAPstart (days)	-	-	-	-15	-6	0	-29	-14	-6	-39	-22	-6
VAPend	Sep 4th	Sep 29th	Oct 16th	Sep 10th	Oct 4th	Oct 20th	Sep 14th	Oct 8th	Oct 24th	Sep 16th	Oct 11th	Oct 27th
Change in VAPend(days)	-	-	-	1	4	11	4	8	17	4	11	26

Supplementary Table 2. Number of days with drought in summer, and frost in winter. Simulated results of JSBACH model using future climate data from five climate models with RPC4.5 and RCP 8.5 for the periods 1981-2010 and 2041-2070

	**1981–2010**		**2011–2040**		**2041–2070**		**2071–2100**	
**Variable**	**South**	**North**	**South**	**North**	**South**	**North**	**South**	**North**
Drought (days) RCP4.5	4	4	13	7	23	9	15	10
Drought (days) RCP8.5	4	4	13	7	23	9	15	10
Soil frost (days) RCP4.5	129	228	104	206	68	189	46	156
Soil frost (days) RCP8.5	134	228	91	199	47	169	25	145

List of abbreviations

Abbreviation	Explanation
C	Carbon
CO_2	Carbon dioxide
CICES	Common International Classification of Ecosystem Services
CMIP5	Coupled Model Intercomparison Project, 5th Phase
ES	Ecosystem Services
GCC	Green Chromatic Coordinate
GCM	Global Climate Model
GPP	Gross Primary Productivity
MAES	Mapping and Assessment of Ecosystem Services
NEE	Net Ecosystem Exchange
PFT	Plant Functional Type
RCC	Red Chromatic Coordinate
RCP	Representative Concentration Pathway
VAP	Vegetation Active Period
